# CRISPR-edited human ES-derived oligodendrocyte progenitor cells improve remyelination in rodents

**DOI:** 10.1038/s41467-024-52444-w

**Published:** 2024-10-09

**Authors:** Laura J. Wagstaff, Nadine Bestard-Cuche, Maja Kaczmarek, Antonella Fidanza, Lorraine McNeil, Robin J. M. Franklin, Anna C. Williams

**Affiliations:** 1 Centre for Regenerative Medicine, Institute for Regeneration and Repair, University of Edinburgh, Edinburgh, UK; 2 Wellcome - MRC Cambridge Stem Cell Institute, Jeffrey Cheah Biomedical Centre, Cambridge Biomedical Campus, University of Cambridge, Cambridge, UK

**Keywords:** Multiple sclerosis, Multiple sclerosis, Regeneration

## Abstract

In Multiple Sclerosis (MS), inflammatory demyelinated lesions in the brain and spinal cord lead to neurodegeneration and progressive disability. Remyelination can restore fast saltatory conduction and neuroprotection but is inefficient in MS especially with increasing age, and is not yet treatable with therapies. Intrinsic and extrinsic inhibition of oligodendrocyte progenitor cell (OPC) function contributes to remyelination failure, and we hypothesised that the transplantation of ‘improved’ OPCs, genetically edited to overcome these obstacles, could improve remyelination. Here, we edit human(h) embryonic stem cell-derived OPCs to be unresponsive to a chemorepellent released from chronic MS lesions, and transplant them into rodent models of chronic lesions. Edited hOPCs display enhanced migration and remyelination compared to controls, regardless of the host age and length of time post-transplant. We show that genetic manipulation and transplantation of hOPCs overcomes the negative environment inhibiting remyelination, with translational implications for therapeutic strategies for people with progressive MS.

## Abstract (web summary)

Increasing age and disease progression negatively impact remyelination in Multiple Sclerosis. Here authors show that genetically edited human oligodendrocyte precursor cells may help overcome remyelination inhibitors and improve remyelination after transplantation into mouse brain.

## Introduction

The later progressive phase of Multiple Sclerosis (MS) is driven by neurodegeneration, and we currently lack effective neuroprotective agents. Neurodegeneration is at least in part due to loss of the myelin sheath around axons (demyelination), leading to disruption of saltatory conduction normally allowing fast action potentials and loss of metabolic support from the oligodendrocyte through the myelin sheath to the axon. In MS, the field is actively exploring ways to therapeutically enhance remyelination to restore this axonal metabolic support for neuroprotection, as although spontaneous remyelination occurs in MS, it becomes less efficient with increasing age, and most people with progressive MS are in mid-life. Although remyelination may in part be driven by surviving oligodendrocytes^1,2^, it is primarily driven by oligodendrocyte progenitor cells (OPCs) present throughout the central nervous system (CNS), which migrate towards areas of demyelination, proliferate and differentiate into myelinating oligodendrocytes, which contact axons forming remyelinated sheaths^3^. OPCs face a variety of inhibitory intrinsic and extrinsic factors in the context of MS that may affect every stage of remyelination, diminishing its efficiency^4^. Most potential therapeutic targets for remyelination have been identified from rodent models of chemically-mediated demyelination where remyelination is more efficient than in human disease because of the young age of animals most frequently used, species differences and a less hostile environment. Clinical trials of pro-remyelinating drugs developed from these models and based on enhancing OPC differentiation have provided the proof of principle that remyelination can be therapeutically enhanced in MS but without yet providing truly effective and reliable regenerative medicines^5–7^.

Therefore, promoting this differentiation step in the remyelination process may not be enough. We know that 30% of MS demyelinated lesions contain low numbers of OPCs^8–10^, suggesting poor recruitment to lesions, and that post-mortem brain samples from people with progressive MS generally have fewer OPCs^11,12^. Ageing contributes to progressive MS, with intrinsic cell and extrinsic environment changes, including reduced OPC recruitment to lesions and differentiation^13–16^, and increased brain stiffness reducing both OPC proliferation and differentiation capacity^17^. MS may accelerate physiological ageing, exacerbating these issues and contributing to cellular senescence^18–20^. This suggests that aged endogenous cells and a hostile environment predispose people with MS to fail to remyelinate.

To circumvent this problem with endogenous ‘aged’ OPCs and the hostile environment in MS and, in addition, due to a lack of cell type-selectivity in most drugs, we revisited the idea of OPC transplants as therapeutic agents. Historically, OPCs, Schwann cells or their precursors and olfactory ensheathing cells^21–23^ were used in preclinical models to successfully achieve myelin replacement, but research into this approach declined, perhaps due to the difficulties in the pragmatics of translation to humans. However, more recent technology has allowed us to generate human (h) OPCs in vitro from stem cells (Embryonic or induced pluripotent stem cells (ESCs/iPSCs)) and to edit these relatively easily using CRISPR/Cas9 technology.

Several studies have shown the ability of transplanted hOPCs to myelinate and remyelinate the CNS of hypomyelinated *Shiverer* mice (*Shi/Shi*), increasing lifespan and improving behavioural outcomes^24,25^, and more recent work has demonstrated the ability of these cells to migrate and remyelinate global models of CNS demyelination^26^. We hypothesised that transplantation of edited stem cell-derived hOPCs, modified to overcome the inhibitory MS environment, may be of even greater benefit. As a proof of concept, we deleted the Neuropilin-1 (NRP1) receptor on hOPCs using CRISPR/Cas9 technology in hESCs, since Semaphorin (SEMA) 3 A is a chemorepulsive factor for OPCs, acting through the NRP1 receptor^27–29^. SEMA3A is highly expressed in human chronic active demyelinated MS lesions, which contain reduced numbers of OPCs^9,28^, and human chronic active lesions are thought to poorly remyelinate^30,31^ and, therefore, are a good target for remyelinating therapies. We have previously shown that increasing SEMA3A expression in rodent demyelinated lesions reduces OPC recruitment and remyelination^9^, thereby modelling MS lesions which fail to remyelinate.

Here, we generated NRP1^−^^/−^ hOPCs and transplanted them into mice either early postnatally or after demyelination in adults, and found enhanced migration into SEMA3A-loaded ‘hostile’ demyelinated lesions and more extensive subsequent remyelination. This work demonstrates the benefit of transplanting edited hOPCs to overcome intrinsic cell and extrinsic challenges of remyelination in the MS context, reigniting OPC transplantation as a potential therapeutic strategy for MS.

## Results

### Generation of membrane-bound GFP hESC line

To test the hypothesis that edited hOPCs could improve remyelination, we generated an hES cell line that constitutively expresses a membrane-targeted green fluorescent protein (GFP) tag (Supplementary Fig. 1A, B)^32^, to easily identify human cells and myelin post-transplantation. Using a standard hESC to oligodendrocyte differentiation protocol (as previously described^33^), we found that GFP was expressed throughout the differentiation process from hES cells (Supplementary Fig. 1B) and into myelin basic protein (MBP) +, OLIG2 + oligodendrocytes (Supplementary Fig. 1C). To examine cell morphology and location in vivo following transplantation, and to confirm we were generating functional human oligodendrocytes, we assessed their ability to myelinate in immunocompromised mice (*Rag2*^*−/−*^)^34^ crossed with *Shiverer (Shi/Shi)* mice which have a mutation in MBP, lacking compact myelin and MBP immunostaining (*Shi/Shi:Rag 2*^*−/−*^)^35^. Thus, human oligodendrocytes and myelin were identified by both the GFP + reporter and MBP + immunofluorescence. GFP + human oligodendroglia were expanded for 1 week in proliferation media following neural sphere dissociation and injected cranially into *Shi/Shi:Rag2*^*−/−*^ pups at P2–P4. 10 weeks post-transplantation, extensive GFP + MBP + myelin was observed throughout the corpus callosum generated by GFP + human oligodendrocytes (Supplementary Fig. 1D, E and Supplementary Video 1). Thus, these human GFP + oligodendroglia are functional and easily located in vivo, even following long-term transplantation.

### Generation of NRP1^−^^/−^ hESC line

To test our hypothesis that editing hOPCs could enhance remyelination, as a proof of principle, we decided to delete Neuropilin-1 (NRP1) from hESC, as the receptor for SEMA3A. SEMA3A is highly expressed in human MS demyelinated lesions at both the transcript and protein level^9,12,28^, especially in chronic active demyelinated lesions which contain few OPCs^9^. Furthermore, when Sema3A protein is overexpressed in mouse demyelinated lesions, there is reduced OPC recruitment and subsequent remyelination due to its chemorepulsive properties^9,28^ and preventing the binding of SEMA3A and NRP1 enhances OPC migration into demyelinated lesions in mouse^9,29^. Thus, the premise was that hOPCs null for NRP1 would be successfully recruited to such lesions and improve remyelination.

**Fig. 1:**
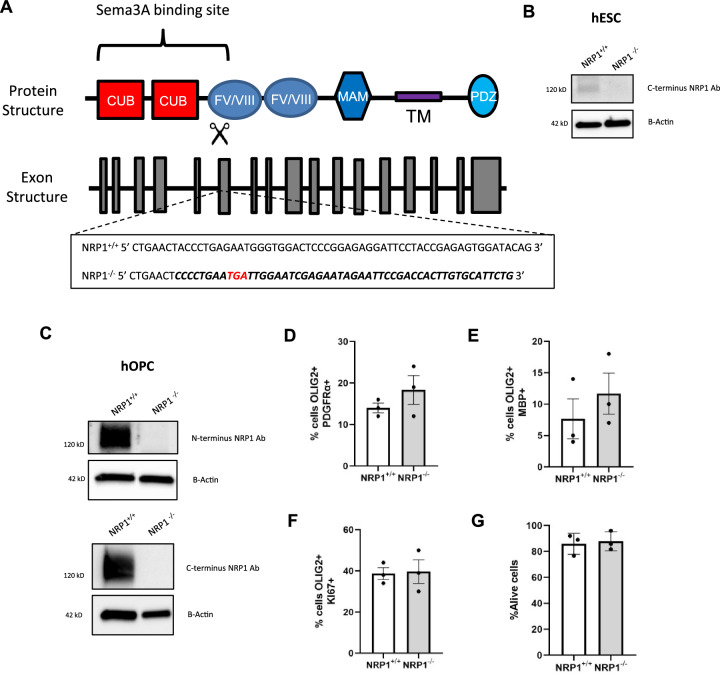
Generation of NRP1-/- line. **A** Diagram of NRP1 protein and genomic structure (Adapted from^29^ and^53^). gRNAs targeting exon 6 induced stop codons (red) and altered the sequence (bold). **B** Western blot of control NRP1^+/+^ and knock out NRP1^−^^/−^ hESC show alterations in the sequence successfully led to the loss of the NRP1 protein. **C** Western blots of NRP1^−^^/−^ hOPCs generated from hESC also show a loss of NRP1. Complete loss of the protein is observed with both the N or C terminus antibodies. Loss of NRP1 does not affect the differentiation of hESC with NRP1^+/+^ and NRP1^−^^/−^ generating equal numbers of PDGFRa+ hOPCs (**D**) and MBP+ oligodendrocytes (**E**), *n* = 3 for all conditions, two-tailed unpaired *t* test, *p* = 0.3027 and *p* = 0.4309 respectively. **F** Proliferation of oligodendroglia was not affected, *n* = 3, two-tailed unpaired *t* test, *p* = 0.8847. **G** No change in cell survival, when measured by flow cytometry using draq7 to assess live/dead cells, was observed, *n* = 3, two-tailed unpaired *t* test, *p* = 0.7699. All graphs plot means ± SEM. Each point represents a separate differentiation culture. Source data are provided in the Source Data file.

We used CRISPR/Cas9 to generate our NRP1 knock-out line in GFP + hESCs with gRNAs targeting exon 6 of NRP1 to generate a complete knock-out of NRP1. Sanger sequencing following RT-PCR demonstrated the insertion of multiple stop codons (Fig. 1A). Successful targeting at the protein level was confirmed by western blot of hESCs (Fig. 1B) and hOPC lysates (Fig. 1C), using both N and C-terminal antibodies to NRP1. To ensure normal karyotype and the absence of big changes between the NRP1^+/+^ and NRP1^−/−^ lines, we performed SNP analysis (Supplementary Data 1). This showed a small number of copy number variants (CNVs) different between the two genotypes, but none of these were in areas predicted as potential gRNA off-targets (Supplementary Data 2). We performed scRNAseq analysis at the differentiation stage when hOPCs are generated, and showed that NRP1^+/+^ and NRP1^−^^/−^ lines were very similar in the proportions of cell types present (Supplementary Fig. 2A–C). Differential gene expression analysis between the NRP1^+/+^ and NRP1^−^^/−^ OPCs was also very similar, with only 5 genes showing differences (Supplementary Fig. 2D–F and Supplementary Table 1, with DEG expression for the other cell types shown in Supplementary tables 2–4). Again, none of these five genes were predicted to be off-targets of the gRNAs. However, the significant reduction in expression of the gene *USP34* (a deubiquitinase) in NRP1^−^^/−^ OPCs, is explained by the SNP analysis with *USP34* in an area lost in NRP1^−^^/−^ cells (Supplementary Fig. 2G).

This deletion of NRP1 also did not negatively impact oligodendroglial generation or behaviour under normal culture conditions as equal numbers of PDGFRα + hOPCs were generated from NRP1^+/+^ and NRP1^−^^/−^ hESCs on differentiation, and both genotypes of cells showed equivalent survival, proliferation and differentiation into MBP + oligodendrocytes (Fig. 1D–G). This demonstrated that NRP1 per se does not play a role in these processes. As NRP1 binds SEMA3A, which is a chemorepulsive signal for OPCs, we next tested the response of NRP1^−^^/−^ hOPCs to SEMA3A.

### NRP1^−^^/−^ oligodendroglia are not affected by the differentiation or chemorepulsive effects of SEMA3A

**Fig. 2:**
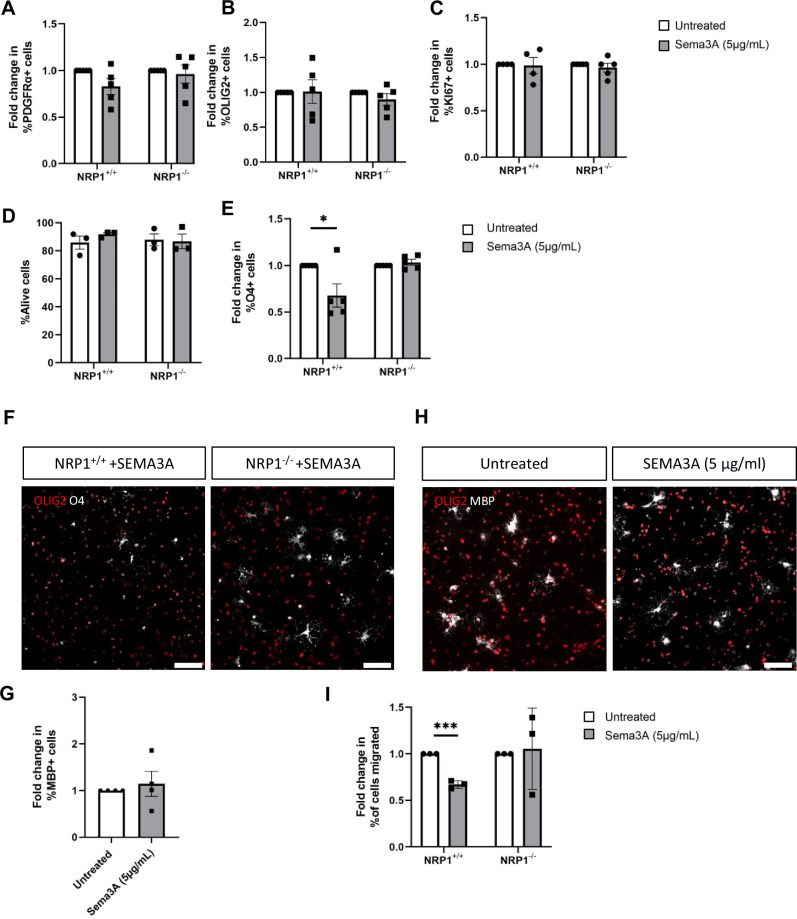
SEMA3A affects early differentiation of oligodendrocytes and inhibits cell migration. NRP1^+/+^ and NRP1^−^^/−^ oligodendroglia were incubated for 3 days with or without 5µg/ml SEMA3A. This did not affect the number of NRP1^+/+^ or NRP1^−/−^ PDGFRα + OPCs (**A**), two-tailed unpaired *t* test, *p* = 0.0798 and 0.7139 respectively, or the number of OLIG2+ oligodendroglia (**B**), two-tailed unpaired *t* test, *p* = 0.9394 and 0.2686 respectively, *n* = 5 for all conditions. **C** Oligodendroglia proliferation was assessed with KI67, and SEMA3A had no effect on proliferation of NRP1^+/+^, *n* = 4, two-tailed unpaired *t* test, *p* = 0.8908 or NRP1^−^^/−^ oligodendroglia, *n* = 5, two-tailed unpaired *t* test, *p* = 0.4602. **D** Flow cytometry of live/dead cells using marker draq7 found SEMA3A did not affect cell survival, *n* = 3 for all conditions, two-tailed unpaired *t* test, NRP1^+/+^ *p* = 0.2814, NRP1^−^^/−^ *p* = 0.8788. **E** A significant decrease in O4 + oligodendrocytes was observed when NRP1^+/+^ differentiating cultures were incubated with SEMA3A, an effect not observed in NRP1^−/−^ oligodendrocytes. *n* = 5 for all conditions, two-tailed unpaired *t* test, NRP1^+/+^ *p = 0.0330, NRP1^−^^/−^ *p* = 0.3706. **F** Representative images showing NRP1^+/+^ and NRP1^−^^/−^ O4 + oligodendrocytes following exposure to SEMA3A, scale bar 100 μm (**G**) NRP1^+/+^ cells were incubated with 5 µg/ml SEMA3A for 1 week. No difference in mature MBP + oligodendrocytes was observed between the treated and untreated groups, *n* = 4 for both conditions, two-tailed unpaired *t* test, *p* = 0.6112. **H** Representative images of MBP + NRP1^+/+^ oligodendrocytes following one-week incubation with SEMA3A, scale bar 100 μm. **I** NRP1^+/+^, and NRP1^−^^/−^ cells were placed in transwells with untreated base media or base media containing 5 µg/ml SEMA3A. Hoechst was used to quantify the total number of cells, and the number of migrated cells was used to calculate the % of migrated cells. SEMA3A reduced NRP1^+/+^ cell migration by 33% but did not affect the migration of NRP1^−^^/−^ cells. *n* = 3 for all conditions, two-tailed unpaired *t* test, NRP1^+/+^ ***p = 0.0002, NRP1^−^^/−^ *p* = 0.8382. All graphs plot means ± SEM. Each point represents a separate differentiation culture. Source data are provided in the Source Data file.

NRP1^+/+^ and NRP1^−^^/−^ differentiating cultures were incubated with 5 µg/ml of recombinant SEMA3A for 3 days (dose chosen as per Syed et al., 2011), with no effect on PDGFRα + hOPC or OLIG2 + oligodendroglia numbers, proliferation or survival (Fig. 2A–D). SEMA3A has previously been shown to decrease rodent OPC differentiation into O4 + oligodendrocytes following short-term incubation in vitro^36^. We similarly found a significant decrease in O4 + cells in the NRP1^+/+^ cells incubated with SEMA3A for 3 days, but this response was absent in our NRP1^−^^/−^ cells suggesting a lack of response to SEMA3A (Fig. 2E, F). To examine the effect of SEMA3A on differentiation to more mature oligodendrocytes, we increased the incubation period to seven days but found no difference in the number of MBP + oligodendrocytes in NRP1^+/+^ cells (Fig. 2G, H), suggesting that SEMA3A does not affect this stage of differentiation of human oligodendrocytes.

As the known function of SEMA3A is chemorepulsion, we next assessed hOPC migration in transwell assays in the presence of SEMA3A. hOPCs were expanded for one week and then plated on transwell membranes for 24 h with or without SEMA3A in the lower chamber. As expected, there was a 30% reduction in NRP1^+/+^ cell migration in response to SEMA3A compared to untreated cells, but this response was not observed in NRP1^−^^/−^cells, which migrated equally in both conditions (Fig. 2I). This suggested that our NRP1^−^^/−^ cells had lost their response to the chemorepulsive effect of SEMA3A in vitro, and so we next tested this in demyelinated lesions in vivo.

### NRP1^−^^/−^ hOPCs migrate towards SEMA3A-loaded demyelinated lesions following transplantation

**Fig. 3:**
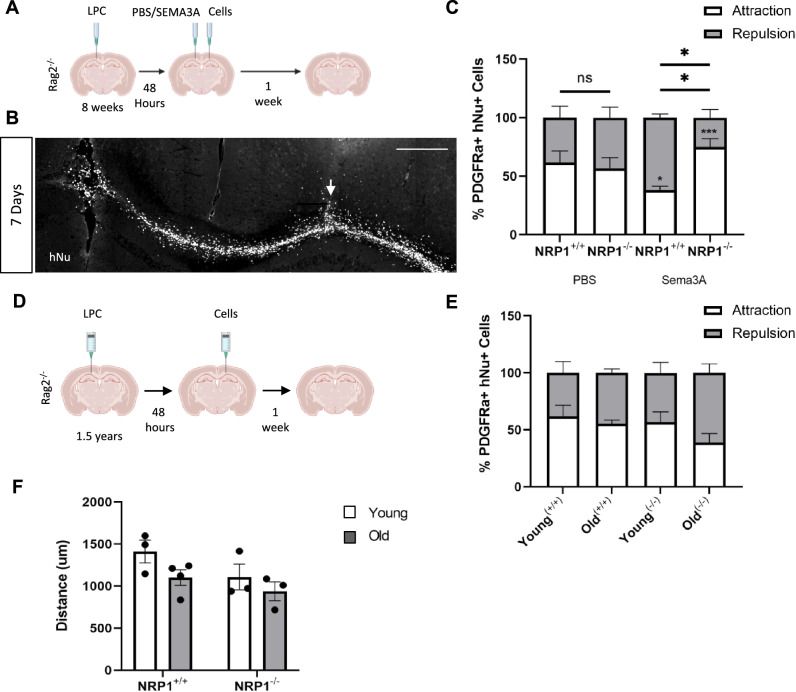
NRP1^−^^/−^ hOPCs migrate towards chronic lesions following transplantation. **A** Diagram of the experiment. A focal demyelinated lesion was made in 8-week-old *Rag2*^−/−^ mice right corpus callosum. After 48 h, the lesion was loaded with PBS or SEMA3A. NRP1^+/+^ or NRP1^−^^/−^ oligodendroglia were injected 2 mm from the lesion site, and cell migration was examined 7 days after when (**B**) hNu+ cells were observed extensively throughout the corpus callosum. Cell migration away/towards the lesion was measured from the injection tract (arrow). Scale bar 400 μm (**C**) The percentage of PDGFRα + hOPCs migrating towards (attraction, white) and away (repulsion, grey) from a PBS or SEMA3A-loaded lesion. When the lesion was loaded with PBS, no significant difference in migration direction was observed between the two cell genotypes with 62% of NRP1^+/+^ hOPCs migrating towards and 38% migrating away from the lesion, *n* = 3, two-away ANOVA, Sidak’s test, *p* = 0.2463 and 57% of NRP1^−^^/−^ hOPCs towards and 43% migrated away from the lesion, *n* = 4, two-away ANOVA, Sidak’s test, *p* = 0.5095. With SEMA3A lesional loading, the majority of NRP1^+/+^ hOPCs migrated away, *n* = 3, two-away ANOVA, Sidak’s test, *p = 0.0309 and the majority of NRP1^−^^/−^ hOPCs migrated towards the lesion, *n* = 3, two-away ANOVA, Sidak’s test, ***p = 0.0004. Comparing NRP1^+/+^ and NRP1^−^^/−^ hOPCs, more NRP1^+/+^ hOPCs migrated away from the lesion *p = 0.0367, whereas more NRP1^−^^/−^ hOPCs migrated towards it *p = 0.0367. Two-way ANOVA, Sidak’s test. **D** Experimental outline. 1.5-year-old adult *Rag2*^−^^/−^ mice received an LPC lesion. 48 h post-lesion, cells were added 2 mm away from the lesion site in the contralateral hemisphere. Analysis was performed one-week post-transplant. **E** The percentage of NRP1^+/+^ (^+/+^) and NRP1^−^^/−^ (^−^^/−^) moving towards (attraction, white and away (repulsion, grey) from the lesion showed no difference between cell genotype or host mouse age. Young^(+/+)^, Old^(−^^/−^^)^ *n* = 3, Young^(−^^/−^^)^, Old^(+/+)^ *n* = 4. Two-way ANOVA with Sidak’s test. **F** The total distance migrated by NRP1^+/+^ and NRP1^−^^/−^ hOPCs from the injection site towards the lesion was equal regardless of host mouse age. NRP1^+/+^ Young, NRP1^−^^/−^ young and old *n* = 3, NRP1^+/+^ old *n* = 4. Two-way ANOVA with Sidak’s test. All graphs plot means ± SEM. Each point represents one mouse and is the average quantification of 3 sections/mouse. Source data are provided in the Source Data file.

To examine the migration of NRP1^−^^/−^ hOPCs into demyelinated lesions in vivo, we needed to use a stereotactically-placed focal model of demyelination to guarantee a precise and known lesion location, with a well-characterised timing of events for remyelination and minimal axonal damage. Therefore, we chose to generate a focal demyelinating lesion in the corpus callosum in the right hemisphere of *Rag2*^*−/−*^ mice, using stereotaxic injection of the demyelinating toxin lysophosphatidyl choline/lysolecithin (LPC), which is consistent in our hands. Sema3A is only transiently expressed in such focal lesions in mice^9^; therefore, after 48 h, the lesion was loaded with recombinant SEMA3A to create a more chemorepulsive environment in the lesion, modelling a chronic active MS lesion. As in our previous published experiments^9^, we injected SEMA3A combined with laminin to maintain its localisation in the lesion site and compared this with the control laminin/PBS injection alone. NRP1^+/+^ or NRP1^−^^/−^ cells were then transplanted 2 mm distant to the lesion, across the midline in the left hemisphere corpus callosum (Fig. 3A). The typical LPC lesion timeline is complete demyelination by 3 days, followed by OPC migration into the lesion and after 10 days, OPC differentiation occurs^37^. At 1 week post-transplant, human cells were found distributed throughout the corpus callosum (Fig. 3B). Cell migration was assessed by the proportion of hOPCs moving towards or away from the lesion from the histologically visible starting point of the injection site. In control demyelinated lesions (PBS, without additional SEMA3A), the majority of hOPCs (60%) migrated towards the lesion, regardless of genotype (Fig. 3C), as expected, as these lesions only have a transient expression of Sema3A and remyelination well. However, most (~ 60%) NRP1^+/+^ hOPCs migrated away from SEMA3A-loaded lesions, in keeping with the known response to the chemorepellent SEMA3A, whereas the majority of NRP1^−^^/−^ hOPCs migrated towards SEMA3A-loaded lesions, demonstrating the loss of response to this chemorepellent (Fig. 3C).

To confirm that this increase in hOPCs near the demyelinated lesion was due to enhanced migration and not simply due to the physical force from the injection, we tracked the transplanted cells 24 h after injection into lesioned *Rag2*^*−/−*^ mice and found that they remained close to the injection tract without spreading into the corpus callosum (Supplementary Fig. 3A). This demonstrates that the distribution of cells we observe in the corpus callosum one-week post-transplant (Fig. 3B) was due to migration of the cells from the injection tract and not a result of the injection itself. Furthermore, hOPCs injected into unlesioned *Rag2*^*−/−*^ mice at the same location in the left hemisphere remained in the injection tract one week after injection and were not observed throughout the corpus callosum (Supplementary 3B). This is similar to previous work demonstrating that transplanted rodent oligodendroglial line cells were only able to migrate in adult rodent spinal cord if the cord was previously irradiated^23^. Therefore, the deletion of NRP1 in hOPCs transplanted into the brain after the demyelinated lesion was generated improved hOPC migration into the lesion made hostile to hOPCs by the addition of the chemorepulsive agent SEMA3A.

As age impacts regenerative capacity (see above), and this is relevant to human translation, we tested the migration capacity of our cells in response to standard LPC lesions in aged animals (with no additional SEMA3A), to determine if the aged environment altered their response). We generated demyelinating LPC lesions in 18-month-old *Rag2*^*−/−*^ mice and transplanted NRP1^+/+^ or NRP1^−^^/−^ cells 48 h later, 2 mm is distant to the lesion, across the midline in the left hemisphere corpus callosum as before (Fig. 3D). Compared to young mice, the OPC responses were very similar, with no difference in the proportion of either NRP1^+/+^ and NRP1^−^^/−^ hOPCs migrating away or towards lesions (overall average of 53.16% (SEM ± 4.978) cells migrating towards lesions) (Fig. 3E). The distance migrated by both NRP1^+/+^ and NRP1^−^^/−^ hOPCs was equal regardless of the age of the host, suggesting that in this context, their intrinsic properties were more important than the environment (Fig. 3F). Therefore, transplanted ES-derived hOPCs migrated equally well in both young and aged animals; an essential quality for transplantation strategies into adult humans. As MS is a chronic condition, with recurring episodes of demyelination, we next tested whether hOPCs after long-term transplantation would still respond effectively to a subsequent episode of demyelination.

### NRP1^−^^/−^ hOPCs transplanted into early postnatal mouse brains migrate into SEMA3A-loaded demyelinated lesions in adulthood

**Fig. 4:**
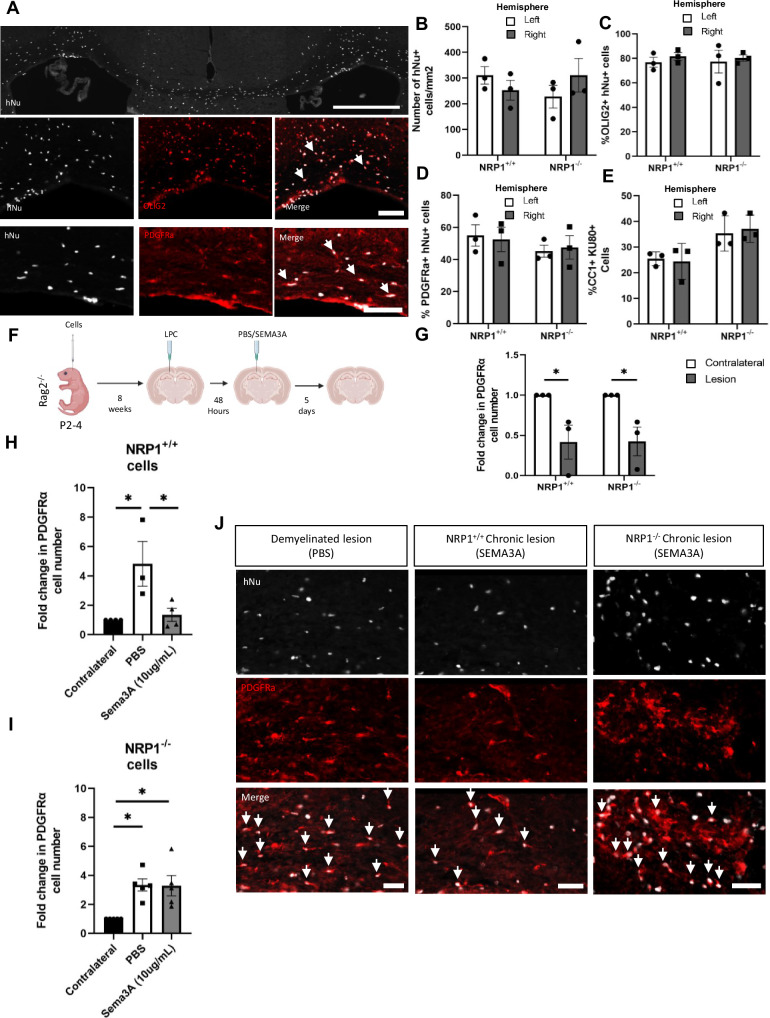
NRP1^−/−^ hOPCs migrate into chronic lesions following long-term transplantation. NRP1^+/+^ or NRP1^−^^/−^ oligodendroglia were injected into P2-4 *Rag2*^−/−^ pups. At 8 weeks, (**A**) hNu + cells were found throughout the corpus callosum. Most hNu + cells were OLIG2 + oligodendroglia and PDGFRα + hOPCs, arrows. Scale bars, top 500μm, bottom 100 μm. **B** There was no difference in NRP1^+/+^ and NRP1^−^^/−^ hNu + cell number between the left/right hemispheres, *n* = 3, two-tailed unpaired *t* test, *p* = 0.3208 and *p* = 0.3486. **C** Most hNu + cells were also OLIG2 + and (**D**) over half were PDGFRα + hOPCs with no difference between cell genotype (OLIG2: *n* = 3, two-tailed unpaired *t* test, *p* = 0.4107 and *p* = 0.7744, PDGFRα *n* = 3, two-tailed unpaired *t* test, *p* = 0.8202 and *p* = 0.7825). **E** NRP1^+/+^ and NRP1^−^^/−^ human cells (identified with KU80^52^) generated similar numbers of evenly distributed CC1-positive oligodendrocytes. *n* = 3, two-tailed unpaired *t* test, *p* = 0.8180 and *p* = 0.7372. **F** Experimental diagram: NRP1^+/+^ or NRP1^−^^/−^ oligodendroglia were injected into P2-4 Rag2^−/−^ pups, a focal LPC-induced demyelinated lesion made in the right corpus callosum at 8 weeks old, loaded with PBS or SEMA3A after 48 h, for cell migration analysis at 5 days. **G** Demyelination reduced the number of NRP1^+/+ ^PDGFRα + hOPCs in the lesion by 59% and NRP1^−^^/−^ PDGFRα + hOPCs by 58% compared to the unlesioned contralateral side at 48 h, (*n* = 3, two-tailed unpaired *t* test, *p = 0.0497 and *n* = 3, two-tailed unpaired *t* test, *p = 0.0324 respectively). **H** At 1 week post-lesion, as expected, there were significantly more NRP1^+/+ ^PDGFRα + hOPCs in PBS-loaded lesions than SEMA3A-loaded lesions (*n* = 3/4, one-way ANOVA, Tukey test, *p* = 0.0309), than the unlesioned contralateral side (*n* = 3, one-way ANOVA, Tukey test, *p = 0.0195), with similar numbers of NRP1^+/+ ^hOPCs in SEMA3A-loaded lesions compared to the contralateral side (*n* = 4, one-way ANOVA, Tukey test, *p* = 0.9362). **I** However, there was an increase in NRP1^−^^/−^ PDGFRα + hOPCs in SEMA3A-loaded lesions compared to the contralateral side, (*n* = 4, one-way ANOVA, Tukey test, *p = 0.0126), similarly to in PBS-loaded lesions, (*n* = 4, one-way ANOVA, Tukey test, *p = 0.0113) with no difference between the 2 lesion conditions (one-way ANOVA, Tukey test, *p* = 0.9980). **J** Representative images of hNu+PDGFRα + hOPCs, arrows, in PBS and SEMA3A-loaded lesions, showing fewer NRP1^+/+^ and more NRP1^−^^/−^ hOPCs in SEMA3A-loaded lesions. Scale bars 50μm. All graphs: means ± SEM. Each point represents one mouse and is the average quantification of 3 sections/mouse. Source data are provided in the Source Data file.

To examine the response of long-term transplanted hOPCs to subsequent demyelination, we established a model where we injected either NRP1^+/+^ or NRP1^−^^/−^ hOPCs intracranially into *Rag2*^*−/−*^ pups at P2-4. We first checked that, 8 weeks post-transplant, our cells had formed an even and integrated population and observed human nuclei spread evenly throughout the corpus callosum with equal distribution in both hemispheres with both hOPC genotypes (Fig. 4A, B). The majority (~ 80%) of these human cells were OLIG2 + oligodendroglia, with over 50% PDGFRα + hOPCs and around 30% CC1 + oligodendrocytes, with the remainder being GFAP + astrocytes (Fig. 4C–E and Supplementary Fig. 4A).

We next made a focal demyelinating lesion in these mice at 8 weeks of age and loaded the demyelinated lesion with PBS (control) or SEMA3A as before (again modelling a chronic demyelinated lesion) (Fig. 4F). The number of hOPCs was significantly depleted at the lesion site (by ~ 60%), regardless of genotype, at 48 h, compared to the uninjured contralateral side (Fig. 4G). This shows that both hOPC genotypes are equally susceptible to LPC-induced demyelination, and so depleted hOPCs must be replaced through migration into the lesion.

hOPC migration was next assessed in these mice one-week post lesion. In response to a control PBS-loaded focal demyelinated lesion, there was a 4-fold increase in lesional NRP1^+/+^ hOPCs compared to the contralateral side. However, in response to a recombinant SEMA3A-loaded lesion, we saw the expected poor recruitment of NRP1^+/+^ hOPCs, with no difference in number between the lesion and the contralateral side (Fig. 4 H, J). In mice that had received early postnatal NRP1^−^^/−^ hOPC transplants, there was a similar increase in NRP1^−^^/−^ hOPC numbers in both control PBS-loaded and SEMA3A-loaded demyelinated lesions (Fig. 4I, J) with successful NRP1^−^^/−^ hOPC migration into demyelinated lesions despite the presence of a chemorepulsive signal and at a time distant to the initial transplant. In case the observed hOPC number difference was due to proliferation rather than migration between the two cell types or lesion environments, we analysed the percentage of KI67 + hNu + human cells in the lesion (Supplementary Fig. 5A), but found no difference in NRP1^+/+^ or NRP1^−^^/−^ cell proliferation in either the PBS or SEMA3A-loaded lesions (Supplementary Fig. 5B). Thus, the change in hOPC number relates to migration, not proliferation.

As endogenous OPC migration is reduced with age in rodents^13^, we explored whether the same was true of our early postnatal long-term transplanted hOPCs with age. We injected NRP1^+/+^ or NRP1^−^^/−^ hOPCs into *Rag2*^*−/−*^ pups at P2-4, aged them to 18 months, generated a demyelinating lesion with LPC and assessed hOPC migration into the lesion after 1 week (Supplementary Fig. 6A). The percentage of NRP1^+/+^ or NRP1^−^^/−^ hOPCs in the lesion was similar in both young and aged mice, again suggesting that ES-generated transplanted hOPCs maintain their capacity to migrate in response to demyelination even after 18 months in an aged mouse brain environment (Supplementary Fig. 6B).

We next tested whether the increase in hOPC migration into chronically demyelinated lesions translated into increased remyelination, as this aids neuroprotection and is the aim of therapeutic benefit.

### Enhanced NRP1^−^^/−^ OPC migration into ‘chronic’ lesions improves remyelination

**Fig. 5:**
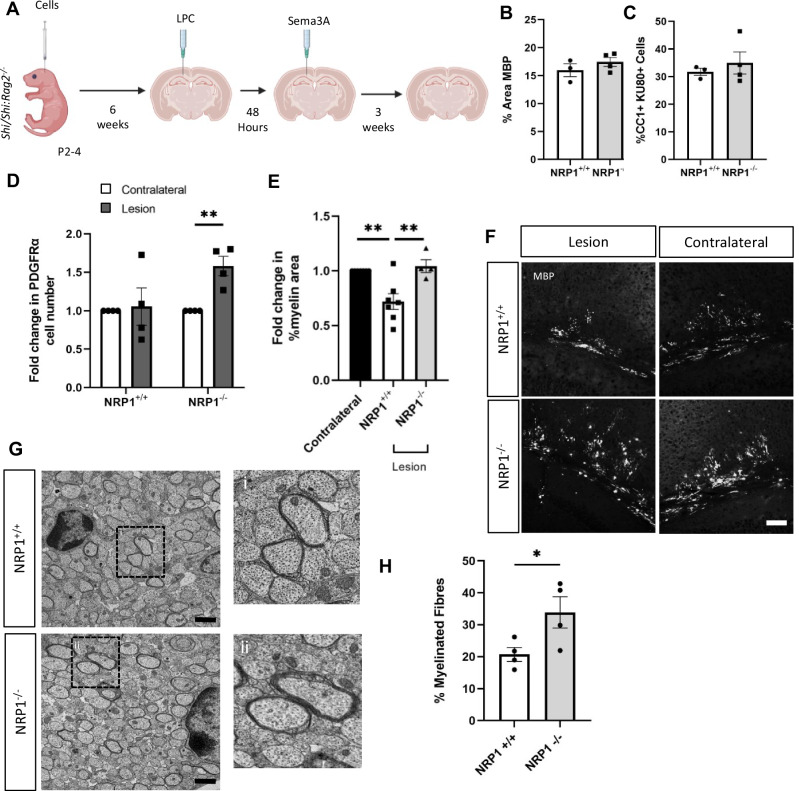
Enhanced migration of NRP1^−^^/−^ hOPCs leads to improved remyelination. **A** Diagram of the experiment. NRP1^+/+^ or NRP1^−^^/−^ hOPCs were injected into P2-4 *Shiverer:Rag2*^*−/−*^ pups, and at 6 weeks of age, an LPC focal demyelinating lesion was made in the right hemisphere in the corpus callosum. After 48 h, the lesion was loaded with SEMA3A and remyelination was assessed after 3 weeks. Both NRP1^−^^/−^ and NRP1^+/+^ hOPCs have equal capacity to myelinate, with no significant difference between (**B**) the % of the area that was MBP + in the corpus callosum of 6 week old mice, *n* = 3 for each condition, two-tailed unpaired *t* test, *p* = 0.3228 or (**C**) the number of CC1 + oligodendrocytes *n* = 3/4 for each condition, two-tailed unpaired *t* test, *p* = 0.5365. **D** one-week post-lesion, there was a significant increase in the number of NRP1^−^^/−^ PDGFRα + hOPCs in the lesion compared to the contralateral *n* = 4, two-tailed unpaired *t* test, **p = 0.0037 but no significant difference in the number of NRP1^+/+^ PDGFRα + hOPCs in the lesion compared to the contralateral. *n* = 4, two-tailed unpaired *t* test, *p* = 0.8262. **E** 3 weeks post-lesion, when NRP1^−^^/−^ cells were present, the % MBP+ area in the lesion site was similar to the contralateral side, n = 4, one-way ANOVA, Tukey test, *p* = 0.8613 but when NRP1^+/+^ cells were present, there was less MBP + area in the lesion *n* = 7, one-way ANOVA, Tukey test, **p = 0.0031. NRP1^−^^/−^ transplants led to significantly more MBP than NRP1^+/+^ transplants, *n* = 4/7, one-way ANOVA, Tukey test, **p = 0.0036. **F** Representative images of MBP immunostaining in lesions and the unlesioned contralateral corpus callosum. Scale bar 100 μm. **G** Representative EM images of the corpus callosum of *Shiverer:Rag2*^*−/−*^ mice with post-natal transplanted NRP1^+/+^ or NRP1^−^^/−^ oligodendroglia, taken 3 weeks post LPC lesion, with higher magnification insets (a and b). Scale bar 1 μm. **H** Quantification of EM images 3 weeks post-lesion showed a significant increase in the % of fibres myelinated with compact myelin (therefore human cell-derived) when NRP1^−^^/−^ oligodendroglia were transplanted, *n* = 4 for each condition, unpaired two-tailed test, *p = 0.0493. Each point represents one mouse and is the average quantification of 3 sections/mouse (**B**–**E**) or ~ 20 images (**H**). All graphs plot means ± SEM. Source data are provided in the Source Data file.

It was difficult to quantify the contribution of human oligodendrocytes to remyelination in the *Rag2*^*−/−*^ mice due to the overwhelming extent of mouse MBP. Instead, we adapted our long-term transplantation model into *Shiverer* mice, which have non-compact myelin lacking MBP^38^, to more easily identify human oligodendrocyte-generated MBP + myelin. We injected NRP1^+/+^ or NRP1^−^^/−^ cells into *Shi/Shi:Rag2*^*−/−*^ mice at P2-P4, which form MBP + human myelin around mouse axons (Supplementary Figs. 1D and 7A) and generated a focal demyelinating lesion, thus affecting human and mouse myelin and oligodendroglia at 6 weeks post-transplant. Lesions were loaded with SEMA3A after 48 h as before, with remyelination assessed 3 weeks later (Fig. 5A). (These are earlier time-points than above as our animal regulations only allow us to retain these transgenic mice until 70 days of age). We first confirmed equal myelination of axons by both NRP1^+/+^ and NRP1^−^^/−^ transplanted hOPCs by assessment of MBP + immunofluorescence per area of the corpus callosum at 6 weeks of age in this model, before demyelination (Fig. 5B) and equal differentiation of these cells into CC1 + oligodendrocytes (Fig. 5C). GFAP + astrocytes were also present and equally distributed between the hemispheres (Supplementary Fig. 7B). At 7 days post-SEMA3A-loaded demyelinated lesion, in the NRP1^−^^/−^ hOPC-transplanted mice, there was an increase in NRP1^−^^/−^ PDGFRa + hOPCs in the lesion, indicating a migration response even with the chemorepulsive cue, which was absent in the NRP1^+/+^ hOPC transplanted mice (Fig. 5D). To determine whether this improved hOPC migration also enhanced remyelination, we analysed the amount of MBP + immunofluorescence in the lesion site at 3 weeks post-lesion compared with the contralateral side. Mice transplanted with NRP1^+/+^ hOPCs had lesions with significantly less MBP signal, whereas lesions in mice transplanted with NRP1^−^^/−^ hOPCs had similar MBP signal compared to the contralateral side, showing more efficient remyelination by these edited human cells (Fig. 5E, F). Ultrastructural analysis of these lesions after 3 weeks found that there were more axons surrounded by compact myelin after transplantation with NRP1^−^^/−^ oligodendrocytes than with NRP1^+/+^ oligodendrocytes (Fig. 5G, H), indicating more remyelination. G-ratio analysis showed no difference in myelin thickness between NRP1^−^^/−^ and NRP1^+/+^ cells or the diameter of the myelinated axons (Supplementary Fig. 7C, D). At 3 weeks, the number of CC1 + cells was equal between NRP1^−^^/−^ and NRP1^+/+^ transplants, suggesting a degree of catch-up in cell number but not myelin produced at this later timepoint (Supplementary Fig. 7E). Remyelinated lesions also had similar numbers of GFAP + astrocytes when compared with the unlesioned contralateral side (Supplementary Fig. 7F). These data demonstrate that in the context of demyelinated lesions hostile to OPC migration, edited hOPCs with improved migration resulted in enhanced remyelination.

## Discussion

Here, we show proof of the principle that hOPCs, edited so that they do not respond to negative environmental signals inhibiting remyelination, improve remyelination in a mouse model. In our experiments, we chose to inhibit the response of hOPCs to SEMA3A, mimicking the increased SEMA3A expression identified in human MS demyelinated lesions that do not remyelinate (chronic active lesions)^9^. The presence of these lesions (as detected by a paramagnetic rim on MR scanning) correlates with a worse prognosis in progressive MS^30,31^. This manipulation improved the recruitment of hOPCs to the SEMA3A-loaded demyelinated lesion and subsequent remyelination. The limitations of this are that we used one protocol for generating hOPCs^33^ with one ES cell line in one focal model of LPC-induced demyelination in the mouse, but this was done to provide proof-of-concept. Vascular endothelial growth factor (VEGF) also binds to NRP1 as well as to its other receptor VEGFR, which dimerises with NRP1. VEGF is an OPC chemoattractant^39^, so the loss of NRP1 may also reduce this signalling.

This work provides evidence for OPC transplantation as a potential therapeutic strategy for MS demonstrating the advantages of transplantation before and after lesions appear. We do not expect that this single manipulation will allow these hOPCs to successfully remyelinate all demyelinated lesions in humans, as there are other inhibitory components. However, this finding allows us to take the next step, to choose other pathways in combination to target and edit, to gain hOPCs that migrate and differentiate in the inhibitory environment of MS lesions, perhaps including Toll-like receptor-2 (TLR-2)^40^ and/or LINGO-1 receptor^41^. Transplanted ES-derived cells may mitigate against the problem of ageing endogenous OPCs, which, at least in rodents, respond less well to both migratory and differentiation cues^13–16^, as we show that hOPCs migrate equally well in both 18-month and 8-week-old mice hosts. If these transplanted stem cell-derived hOPCs also behave similarly to younger OPCs in their response to pro-differentiating pro-remyelinating compounds, even in an aged environment (as they do in vitro^42,43^), then combining cell transplantation and this class of compounds may be an optimal treatment route for MS.

A hurdle for cell therapy in neurological disease is how to safely and effectively transplant cells into the human CNS. Transplanted cells must be able to reach lesions in order for them to be beneficial. Recent clinical trials have shown safe transplant of neural progenitor cells into MS patients through intrathecal injection^44^, intraventricular injection^45^ and intranasal transplant of OPCs and mesenchymal stem cells is successful in rodents^46,47^. Here, we have shown our edited hOPCs are able to migrate through brain parenchyma under lesion conditions travelling 2 mm by one-week post-transplant, demonstrating they are able to migrate to lesions distal to their site of the transplant. Following long-term transplantation, cells reach both the anterior and posterior of the corpus callosum as well as in deep white matter tracts such as the anterior commissure (Supplementary Fig. 8A). When placed into the ventricular system, these cells also survive and distribute through the CSF channels (Supplementary Fig. 8B), providing a potential alternative transplantation route. Transplanted rodent oligodendroglial cell line cells are reported to migrate in irradiated rodent spinal cord but not in normal tissue^23^, whereas here, we (as well as others^26^) have shown that hOPCs are able to populate the brain in immunosuppressed mice in development and in response to demyelination. This highlights that cell type and context are important.

Gene-edited cell transplantation is already used in studies in other human diseases, such as inducible c-MYC expressing neural stem cells promoting their growth in large vessel stroke patients^48^, stem cell-derived pancreatic islet cells edited to evade the immune response in type 1 diabetes mellitus (ClinicalTrials.gov Identifier: NCT04678557) and CRISPR-edited haematopoietic stem cells (HSCs) to correct mutations in the *Arylsulfatase-A* (*ARSA*) gene in metachromatic leukodystrophy^49^. Furthermore, recently, mouse HSC transplants engineered to overexpress Sema3F were tested in a preclinical mouse model of brain demyelination, leading to an increase in Sema3F-expressing monocytes/macrophages in the lesion, which, unlike Sema3A, is a chemoattractant for OPCs, and leading to increased OPC recruitment and accelerated remyelination^50^.

The technology of producing many hOPCs from stem cells with accurate and relatively easy gene editing makes this concept of therapeutic cell transplantation in human MS more realistic. This approach could potentially be used to circumvent the issue of aged cells and a hostile environment for repair, providing a selective effect and a potential therapeutic strategy for the future.

## Methods

### In vitro differentiation of human oligodendroglia from hESC

GFP, NRP1^+/+^ and NRP1^−^^/−^ hESCs were cultured on human recombinant laminin-521 coated plates (5 μg/ml, Biolamina) in StemMACS™ iPS-Brew XF, human medium (Miltenyi Biotec Inc.) with 1% Antibiotic Antimycotic Solution (Sigma). Once confluent, hESCs were differentiated into oligodendroglia using a previously published protocol^33^. Briefly, hESCs were lifted with accutase and resuspended in StemMACS™ iPS-Brew XF, 1% Antibiotic Antimycotic Solution and ROCK inhibitor y-276321 (10 µM, Tocris). 5 × 10^6^ cells were seeded per well in AggreWell™400 microwell culture plates (Stem cell technologies) and cultured overnight to form embryoid bodies. Embryoid bodies were mechanically lifted, transferred to a rotatory shaker and cultured in neutralisation media. Neuralisation media consists of chemically defined media (50% F12 (Invitrogen), 50% Iscove’s modified Dulbecco’s medium (Invitrogen), 1% chemically defined Lipid 100 (Invitrogen), BSA (5 mg/ml, Sigma), monothioglycerol (450 μM, Sigma), 1% Antibiotic Antimycotic Solution (Sigma), insulin (7 mg/ml, Roche), transferrin (15 mg/ml, Roche)), supplemented with activin inhibitor SB 431542 (10 μM, Sigma), N-acetyl cysteine (1 mM, Sigma) and LDN193189 (0.1 µM Stratech). After 10 days, the neural spheres were caudualised in a chemically defined medium supplemented with N-acetyl cysteine (1 mM, Sigma), heparin (5 μg/ml, Sigma), retinoic acid (0.1 μM, Sigma) and basic fibroblast growth factor (FGF-2) (10 ng/ml, PeproTech) for 7 days. Cell morphology was used to assess neural conversion when cells were plated on laminin-coated plates (10 μg/ml, L2020, Sigma) overnight. Neuralized spheres were picked and transferred to advanced DMEM (Invitrogen), containing 0.5% GlutaMAX (Invitrogen), 1% N2 (Invitrogen), 1% B27 (Invitrogen), heparin (5 μg/ml, Sigma) and 1% Antibiotic Antimycotic Solution (Sigma). Neural spheres were ventralized by supplementing the media with purmorphamine (1 μM, Calbiochem), retinoic acid (1 μM, Sigma) and FGF-2 (10 ng/ml, PeproTech), for 7 days. FGF2 was then withdrawn from this media for 2 weeks. hOPC proliferation was promoted by reintroducing FGF2 (10 ng/ml) and supplementing the media with T3 (60 ng/ml, Sigma) PDGFα (20 ng/ml, PeproTech), SAG (1 μM, Calbiochem), purmorphamine (1 μM, Sigma) and IGF-1 (10 ng/ml, PeproTech). Spheres were dissociated after 2 weeks using the Worthington papain dissociation system as per the manufacturer’s instructions. 4 × 10^4^ cells were plated in 40 µl drops on coverslips coated with poly-ornithine (1:100, Sigma), laminin (10 μg/ml, Sigma) Fibronectin (20 μg/ml, Sigma) and Matrigel (Corning). 1 × 10^6^ cells were plated per well on coated 6 well plates. hOPCs were maintained in proliferation media or differentiated into oligodendrocytes by culturing for 1 week in advanced DMEM (Invitrogen), containing 0.5% GlutaMAX (Invitrogen), 1% N2 (Invitrogen), 1% B27 (Invitrogen), heparin (5 μg/ml, Sigma) and 1% Antibiotic Antimycotic Solution (Sigma) supplemented with IGF-1 (10 µg/ml, PeproTech), T3 (60 µg/ml, Sigma) and ITS (Insulin-transferrin-sodium selenite, 1:100, Sigma).

### Generation of GFP hESC line

Membrane-tagged GFP+ hESCs were generated using zinc-finger recombinase in the RC17 line as previously described^32^. Briefly, GFP was tagged using the palmitoylation sequence of GAP43. This 60 bp sequence from the N-terminal of GAP43 is associated with its membrane attachment. The membrane-targeting GFP construct was then inserted at the AAVS1 locus using the pZDonor-AAVS1 Puromycin Vector Kit (Sigma) in conjunction with the CompoZr Targeted Integration Kit (Sigma) as per the manufacturer’s instructions. Puromycin selection was used to establish positive clones. DNA was extracted using a DNeasy blood and tissue kit (Qiagen) as per the manufacturer’s instructions, and Sanger sequencing (Source Bioscience) was used to confirm insertion. Plasmid sequence can be found^32^ and mapped (Supplementary Fig. 1A), generated using SnapGene® software.

### Generation of NRP1^−^^/−^ knockout line using Alt-R™ CRISPR-Cas9 genome editing system

gRNA sequences were designed with chopchop (https://chopchop.cbu.uib.no/) to target exon 6. gRNA sequence: CGGGAGTCCACCCATTCTCA, was compared on BLAST to confirm specificity to NRP1. Alt-R® CRISPR-Cas9 crRNAs (IDT) were then generated from this sequence: /AltR1/rCrG rGrGrA rGrUrC rCrArC rCrCrA rUrUrC rUrCrA rGrUrU rUrUrA rGrArG rCrUrA rUrGrC rU/AltR2/. gRNA complexes (crRNA:tracrRNA) were generated by annealing crRNAs with Trans-activating crRNA (tracrRNA) (IDT). Briefly, 1.2 μl of 100pmoles/μl crRNA was mixed with 1.2 μl of 100pmoles/μl tracrRNA and then annealed using a Bio-rad PCR machine (Programme: 95 °C: Normal ramp rate 3–6 degrees/sec, 95 to 25 °C: Ramp rate 0.1 degrees/sec, 25 to 4 °C: Ramp rate 0.5 degrees/sec). The Cas-9 gRNA complex was then formed by incubating 10 µg of Alt-R S.p. Cas9 Nuclease V3 (IDT) with the crRNA:tracrRNA complex for 10 min at room temperature before plunging into ice. hESCs were incubated with 10 µM ROCK inhibitor for 2 h then lifted with accutase. Cells were transfected using a P3 Primary Cell 4D-Nucleofector™ X Kit (Lonza) as per the manufacturer’s instructions. Clones were picked and expanded on laminin-521 coated plates in StemMACS™ iPS-Brew XF. DNA was extracted using a DNeasy blood and tissue kit (Qiagen) as per the manufacturer’s instructions, and results from Sanger sequencing (Source Bioscience) were analysed by TIDE to confirm indels at the cut site^51^. mRNA was extracted from selected clones using an RNeasy Mini Kit (Qiagen) as per the manufacturer’s instructions. RT-PCR amplified exons 3–8, and the PCR products were sent for Sanger sequencing (Source Bioscience). Snapgene software (from Insightful Science; available at snapgene.com), was used to compare clone sequences to control, confirming the insertion of a stop codon at exon 6 and 7. The absence of protein was confirmed by western blot. Cells were used at passage number 55 for NRP1^+/+^ cells and 59 for NRP1^−^^/−^ and verified using SNP analysis to ensure they were karyotypically normal and otherwise similar. SNP array experiments were performed using the CytoSNP 850 K BeadChip from Illumina and analysed in GenomeStudio 2.0 with the plug-in cnvPartition 3.2.0. The CNVs obtained were filtered to focus on the ones present in NRP1^−^^/−^ but not in the NRP1^+/+^ cells. The genes from the 37 areas affected were examined, and their expression was checked with the transcriptomics data as well as cross-referenced with potential off-targets of the gRNA (Supplementary Fig. 2G, Supplementary Data 1 and Supplementary Data 2).

### Single-cell RNAseq

Single cells from human NRP1^+/+^ and NRP1^−^^/−^ ES cells differentiated to transplant-ready stage were processed through the Chromium Single Cell Platform using a Chromium Next GEM Single Cell 3′ GEM Library and Gel Bead kit (v.3.1 chemistry, PN-1000121, 10x genomics) and a Chromium Next GEM ChipG kit (PN-1000120) and processed following the manufacturer’s instructions. Libraries were sequenced using a NovaSeq 6000 sequencing system (PE150 (HiSeq), Illumina). Alignment to the reference genome, feature counting and cell calling were performed following the 10x Genomics CellRanger (v.7.0.0) pipeline with the human reference genome provided by 10x - refdata-gex-GRCh38-2020-A. The downstream analysis was performed in R v4.2.1, with the QC performed with scatter, using the outliers from the isOutlier function for the library size filtering and a threshold of 6% of mitochondrial genes. A total of 8157 cells from NRP1^−^^/−^ and 8948 cells from NRP1^+/+^ were kept for downstream analysis. The normalisation, feature variance estimation and dimensional reduction were performed with scran, with logNormCounts(), modelGeneVar() and runPCA()/runTSNE() respectively. Clustering was performed with Seurat’s implementation of the Louvain clustering, and the clusters were annotated using canonical markers and lists of differentially expressed markers between clusters obtained with wilcoxauc() from the immunogenomics /presto package. Oligodendroglia was defined as all the clusters positive for OLIG2, astrocytes as the clusters positive for AQP4, two clusters were classified as proliferating as they had high levels of G2M genes, such as CDK1 and the two remaining clusters were labelled as CNS cells as we believe these are less differentiated cells, with an absence of specific markers in the differentially expressed genes (all potential marker genes were widely expressed in the whole dataset). A subcluster of the oligodendroglia with the highest PDGFRA expression was labelled as OPCs. A Differential Expression analysis between NRP1^−^^/−^ and NRP1^+/+^ cells was performed on oligodendroglia, OPCs and astrocytes with MAST using Seurat’s FindMarkers() wrapper with the default thresholds of 0.25 logFC and 0.1 pct cells expressing the gene. The raw data and processed counts and metadata are available at GEO under the accession number GSE241451.

### Immunofluorescence

Specific antibody information can be found in Supplemental Table 5. Where necessary, human cells were identified with human nuclear marker^24^ (hNu) (Millipore) or KU80^52^ (Cell signalling).

### Tissue

Mice were perfused with 4% paraformaldehyde (PFA) under terminal anaesthetic. Brains were dissected and fixed in 4% PFA overnight at 4 °C before being dehydrated in increasing concentrations of sucrose (15%, 30%), over 3 days at 4 °C. Brains were frozen whole in 2-methyl-butane. Cryosections were cut at 16 μm and collected serially. Cryosections were washed in PBS and then blocked in PBS containing 10% horse serum (v/v) and 0.1% triton-X (v/v) for 1 h at room temperature. When mouse primary antibodies were used, a mouse on mouse (M.O.M) blocking kit (Vector labs) was also added at 1:40. Primary antibodies were incubated overnight at 4 °C in the blocking solution. The following day, sections were washed and incubated with appropriate Alexa secondary antibodies (1:1000, Thermofisher) and Hoechst (1:1000, Sigma) in PBS for 1 h at room temperature. Sections were washed in PBS and mounted with Fluoromount (Southern Biotech).

### Cells

Coverslips were washed with PBS and then fixed for 10 min in 4% PFA. Coverslips were washed in PBS and then blocked in PBS containing 10% horse serum (v/v) and 0.1% triton-X (v/v) for 1 h at room temperature. Primary antibodies were incubated overnight at 4 °C in the blocking solution. The following day, coverslips were washed and incubated with appropriate Alexa secondary antibodies (1:1000) and Hoechst (1:1000) in PBS for 1 h at room temperature. Coverslips were washed in PBS and mounted with Fluoromount (Southern Biotech).

Imaging was performed on a wide-field Zeiss observer and a Leica TCS SP8 confocal.

### Mice

All work was carried out to the standard UK Home Office regulations under project licences PADF15B79 and PP1335335. Mice were housed in groups no larger than 5 in individually vented cages with unrestricted access to food and water on a 12-h light/dark cycle. The majority of experiments used homozygous *Rag2*^*−/−*^ mice (C57/Bl6J background)^34^ (Jackson Labs). Remyelination studies were performed using *Shiverer (Shi/Shi:Rag2*^*−/−*^) mice (C3HeB/FeJ x C57/Bl6J background) (Jackson Labs). Both male and female mice were used in experiments.

### Longer-term cell transplantation

Female mice were exposed to isoflurane daily when showing signs of pregnancy to acclimatise them to the smell and avoid pup rejection after transplant operations using this anaesthetic. hOPCs were dissociated from neural spheres as described above and resuspended in proliferation media. A 33-gauge Hamilton™ Neuros™ syringe was used to inject 2.5 × 10^5^ cells in 2 μl volume bilaterally at a depth of 2 mm into the developing corpus callosum in P2-4 pups, anaesthetised with isoflurane. Pups recovered on a heat pad, before being wrapped in the nest bedding and returned to the cage.

### LPC-induced focal demyelinating lesion

6 week old *Shiverer:Rag2*^*−/−*^ mice, 8 week old *Rag2*^*−/−*^ mice or 18 month old *Rag2*^*−/−*^ mice were anaesthetised with isoflurane. Vetergesic and rimadyl were given for analgesia. A sagittal incision was made to expose the skull. Using a stereotactic rig, a 0.4 mm dentist drill bit was used to drill a hole in the right hemisphere 1.2 mm anterior posterior (AP), 1 mm lateral (L) from the bregma. A 33-gauge Hamilton™ Neuros™ syringe filled with LPC (10 mg/ml) was inserted at a depth of 1.4 mm. 2 μl LPC was dispensed at a rate of 0.5 μl/minute. The syringe was held in place for a further 3 min to prevent backflow before suturing. 48 hours later, the incision was reopened. A stereotactic rig was used to guide a 33-gauge Hamilton™ Neuros™ syringe to the lesion coordinates, and the lesion was loaded with 2 μl PBS or Sema3A (10 μg/ml) combined with 10 μg/ml laminin at a rate of 0.5 μl/minute (as in ref. ^9^). The syringe was held in place for 3 min to prevent backflow before resuturing.

### Short-term cell transplantation

hOPCs were cultured in proliferation media for one week. Cells were detached with accutase and resuspended in proliferation media. 48 hours after the creation of a focal demyelinating lesion, the incision was reopened. Using a stereotactic rig, a 0.4 mm dentist drill bit was used to drill a hole in the left hemisphere 1.2 mm anterior-posterior, − 1 mm lateral from the bregma. 2.5 × 10^5^ cells in 2 μl were injected at a depth of 1.4 mm using a 33-gauge Hamilton™ Neuros™ syringe. The syringe was held in place for 3 min to prevent backflow before resuturing.

### Western blotting

Cells were washed with PBS and lysed with RIPA buffer containing protease and phosphatase inhibitors. Protein concentrations were determined using a BCA kit as per the manufacturer’s instructions.

Protein samples were diluted in RIPA buffer and 5x Laemmli buffer to a working concentration of 1x. 10 µg of protein was loaded per well on a 4–15% precast polyacrylamide gel (Biorad) and run at 100 mV for 1 h. A wet transfer system (Biorad) was used at 400 mA for 2 h to transfer the protein onto a PVDF membrane. Membranes were stained with Ponceau S (Sigma Aldrich) to confirm the successful transfer of proteins and blocked for 1 h at room temperature in 5% milk/TBS-T. Primary antibodies were incubated overnight at 4 °C on a rotatory shaker in 5% milk/TBS-T. Membranes were washed with TBS-T and incubated with the appropriate HRP-conjugated secondary antibodies in 5% milk/TBS-T for 1 hour at room temperature. For signal detection, membranes were incubated with SuperSignal™ West Femto Maximum Sensitivity Substrate (Thermofisher), as per the manufacturer’s instructions. Beta-actin was detected using Pierce™ ECL Plus western blotting Substrate. Membranes were visualised using a ChemiDoc MP Imaging System (Biorad).

### Flow cytometry

hOPCs were cultured in differentiation media with or without SEMA3A (5 μg/ml) for 72 h. They were then lifted with accutase and resuspended in PBS with approximately 5 × 10^4^ cells per 100 µl. Samples were stained with DRAQ7™ (1:1000, Abcam) to detect dead and membrane-compromised cells. Samples were analysed using The NovoCyte Advanteon flow cytometer (Agilent Technologies, Inc. 2021) with the NovoExpress 1.5.0 software (Agilent Technologies, Inc. 2021) to determine live/dead cell populations. The gating strategy is shown in Supplementary Fig. 9.

### Transwell assays

Neurospheres were dissociated, and oligodendroglia expanded in proliferation media for 1 week. 8 μm pore transwells (Corning) were set up with base media (advanced DMEM, 0.5% GlutaMAX, 1% N2, 1% B27, heparin (5 μg/ml) and 1% Antibiotic Antimycotic Solution) with or without Sema3A (5 μg/ml) in the lower chamber. Oligodendroglia were lifted with accutase and resuspended in base media. 4 × 10^4^ cells were plated in the top chamber of the transwell. After 24 h, both sides of the membrane were fixed in 4% PFA and stained with Hoechst (1:1000). Whole membranes were imaged on a Zeiss observer. The cells on the top of the membrane were then removed by cell scraping and the membrane was reimaged to capture only the migrated cells on the bottom of the membrane. The total number of cells and the number of migrated cells were quantified to calculate the percentage of cells migrated.

### Electron microscopy

Mice were transcardially perfused with PBS, followed by 4% PFA (w/v) and 2% glutaraldehyde (v/v; TAAB Laboratories) in 0.1 M phosphate buffer. The tissue was fixed overnight at 4 °C then transferred to PBS. The lesion site in the corpus callosum was isolated and post-fixed in 1% osmium tetroxide. Samples were then dehydrated and embedded in Araldite resin blocks. Ultrathin 60 nm sections were cut and stained with uranyl acetate and lead citrate. Grids were imaged on a JEOL transmission electron microscope. Fields of view were randomly selected with ~ 20 images taken per animal. Axons were counted in Fiji from ~ 20 fields of view per mouse. g-ratios were measured using Qupath software, analysing ~ 100 axons per mouse.

### Statistical and image analysis

The experimenter was blinded to the experimental conditions during imaging and analysis of data. An ImageJ macro was used to blind image titles. Image analysis was carried out using Fiji/ImageJ (v1.54) and QuPath (v0.3.1) software. Manual cell counts were performed using both programmes. Where cell numbers are normalised, reporting fold-changes, in vivo this is done with absolute cell numbers. In vitro, this is done with cell percentages. MBP percentage area was analysed using ImageJ. Statistical analysis was performed using GraphPad Prism (8.3.0). Specific statistical tests are described in all figure legends and *p*-values reported. Significance was set as *p* < 0.05*, *p* < 0.01** and *p* < 0.001***. All graphs are representative of means ± SEM. Each individual point represents (i) a different mouse, which is an average analysis from 3 serial sections, or (ii) a separate cell culture differentiation, which is an average of counts from 4 fields of view per coverslip. For flow cytometry and transwell experiments, each point represents a separate cell culture differentiation.

Illustrations created with BioRender.com.

### Reporting summary

Further information on research design is available in the Nature Portfolio Reporting Summary linked to this article.

## Supplementary information

Supplementary Information

Peer Review File

Description of Additional Supplementary Files

Supplementary Data 1

Supplementary Data 2

Supplementary Video 1

Reporting Summary

## Source data

Source data

## Data Availability

The single-cell RNA sequencing raw data and processed counts and metadata are available at GEO under the accession number GSE241451. The data generated in this study are provided as a Source Data file. Source data are provided in this paper.
