# Supplementary material for: CRISPR-edited human ES-derived oligodendrocyte progenitor cells improve remyelination in rodents
Source: Nat Commun. 2024 Oct 9;15:8570. doi: 10.1038/s41467-024-52444-w (PMC11464782; doi:10.1038/s41467-024-52444-w)
Supplement: Supplementary file 1 — Supplementary Information [file 41467_2024_52444_MOESM1_ESM.pdf]

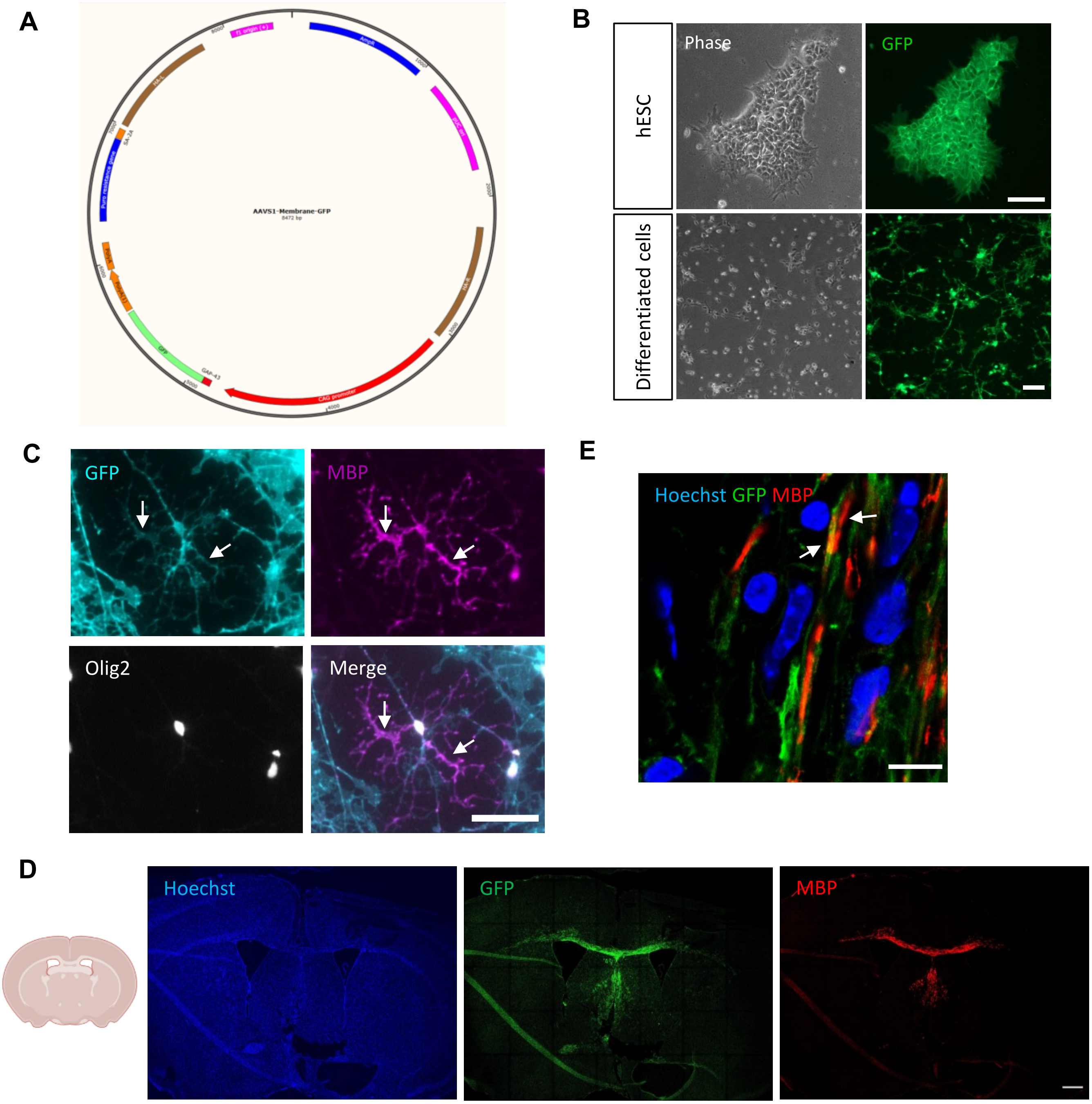
A
AAVS1-Membrane-GFP
8472 bp
f1 origin (+)
AmpR
pUC ori
HA-R
CAG promoter
GAP-43
GFP
PolyA(1)
PolyA
Puro resistance gene
SA-2A
HA-L
1000
2000
3000
4000
5000
6000
7000
8000
B
hESC
Differentiated cells
Phase
GFP
C
GFP
MBP
Olig2
Merge
E
Hoechst GFP MBP
D
Hoechst
GFP
MBP

Supplementary Figure 1 The generation of a membrane-bound GFP tagged hESC line. (A) Diagram showing plasmid map. (B) hESC express membrane-bound GFP tag which is maintained following oligodendroglial differentiation. Scale bars 50µm. (C) GFP+ MBP+ oligodendrocyte. MBP+ processes are GFP+, arrows. Scale bar 50µm. (D) GFP+ oligodendroglia were injected bilaterally into the developing corpus callosum of p2-4 *Shi/Shi:Rag2*-/- mice. Coronal sections, see diagram for orientation, taken 10 weeks following transplantation show GFP+ cells have migrated throughout the corpus callosum and generated MBP+ myelin sheaths. Scale bar 500µm. (E) Still image taken from Supplementary Video 1. Human GFP+ (green) oligodendrocytes generating MBP (red) (arrows) 10 weeks after transplantation into *Shi/Shi:Rag2*$^{-/-}$ mice. Hoechst (blue). Scale bar 10µm

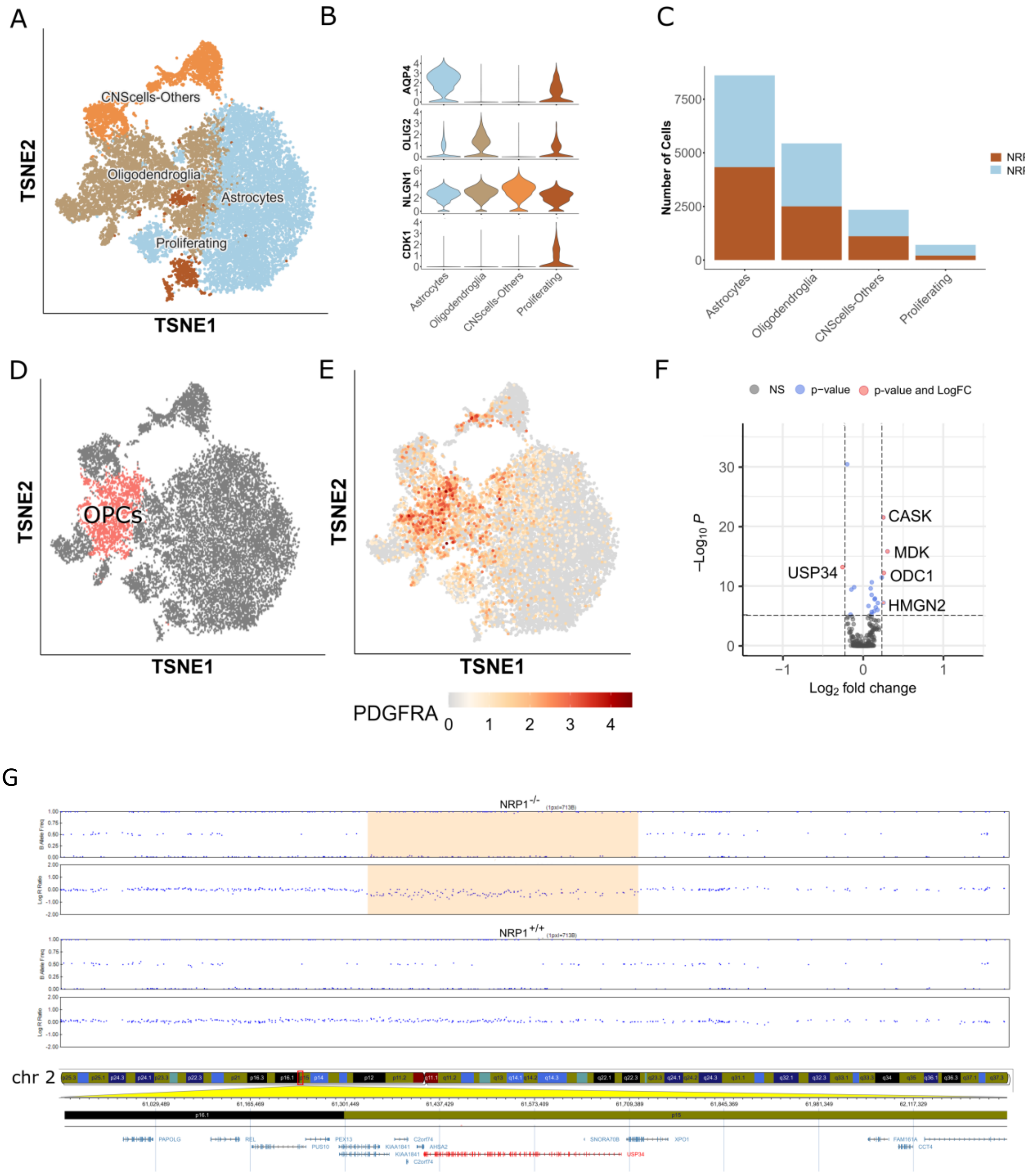

A
CNScells-Others
Oligodendroglia
Astrocytes
Proliferating
TSNE2
TSNE1
B
AQP4
OLIG2
NLGN1
CDK1
Astrocytes
Oligodendroglia
CNScells-Others
Proliferating
C
Number of Cells
7500
5000
2500
0
NRP1+/+
NRP1-/-
Astrocytes
Oligodendroglia
CNScells-Others
Proliferating
D
OPCs
TSNE2
TSNE1
E
TSNE2
TSNE1
PDGFRA
0 1 2 3 4
F
NS
p-value
p-value and LogFC
−Log10 P
30
20
10
0
CASK
MDK
ODC1
USP34
HMGN2
−1 0 1
Log2 fold change
G
NRP1-/-
NRP1+/+
chr 2
PAPOLG
REL
PUS10
PEX13
KIAA1841
C2orf74
AHSA2
USP34
SNORA70B
XPO1
FAM161A
CCT4

Supplementary Figure 2 Characteristics of transplanted cells (A-F) single-cell RNAseq bioinformatics analysis of human cells at the point of transplantation, with (A) Dimensional reduction representation (TSNE) with cells coloured according to the main cell-types detected. (B) Violin plot showing canonical genes for the different cell-types. (C) Proportions of $NRP1^{+/+}$ and $NRP1^{-/-}$ of each cell-type. (D) Cluster of OPCs used for downstream analysis, defined as the cluster with the highest expression of PDGFRA, with (E) showing a feature plot with superimposed expression of PDGFRA. (F) Volcano plot showing the differentially expressed genes between $NRP1^{+/+}$ and $NRP1^{-/-}$ OPCs. Positive values show gene transcripts upregulated in $NRP1^{-/-}$ cells. (G) SNP array analysis of an area of chromosome 2 which is deleted in $NRP1^{-/-}$ cells which incorporates the gene *USP34* and likely accounts for its lower gene expression.

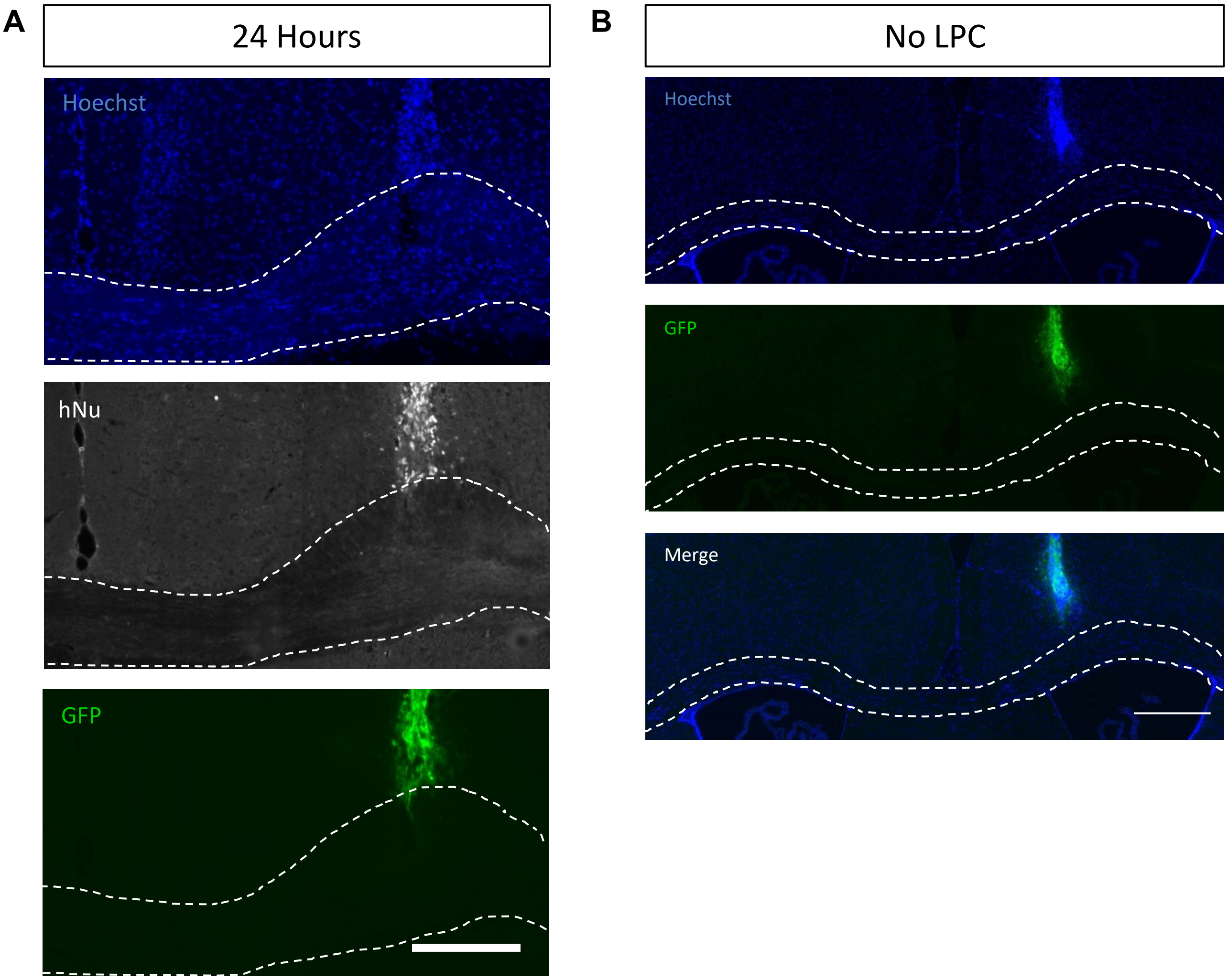

Supplementary Figure 3. Cell movement requires a stimulus that is not the result of injection forces. (A) 24 hours after transplant the human cells largely remain in the injection tract and do not enter the corpus callosum (highlighted between dotted lines), hNu (white), Hoechst (blue). Scale bar = 250μm. (B) At 1 week post transplant, when no demyelinating lesion is present, cells remained in the injection tract and did not enter the corpus callosum (highlighted between dotted lines). GFP (green), Hoechst (blue). Both from representative images of 3 mice. Scale bar = 500μm.

A

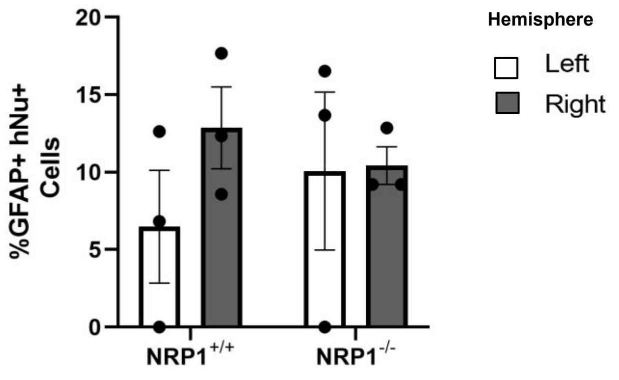

Supplementary Figure 4: Uninjured rag 2 mice 6 weeks post transplants. $NRP1^{+/+}$ and $NRP1^{-/-}$ cells generate equal numbers of astrocytes that are evenly distributed in both hemispheres. n=3 for each condition, two-tailed unpaired t-test, p=0.2296 and p=0.5982. Each point represents 1 mouse, and is the average quantification of 3 sections/mouse. Graph plots: means ± SEM.

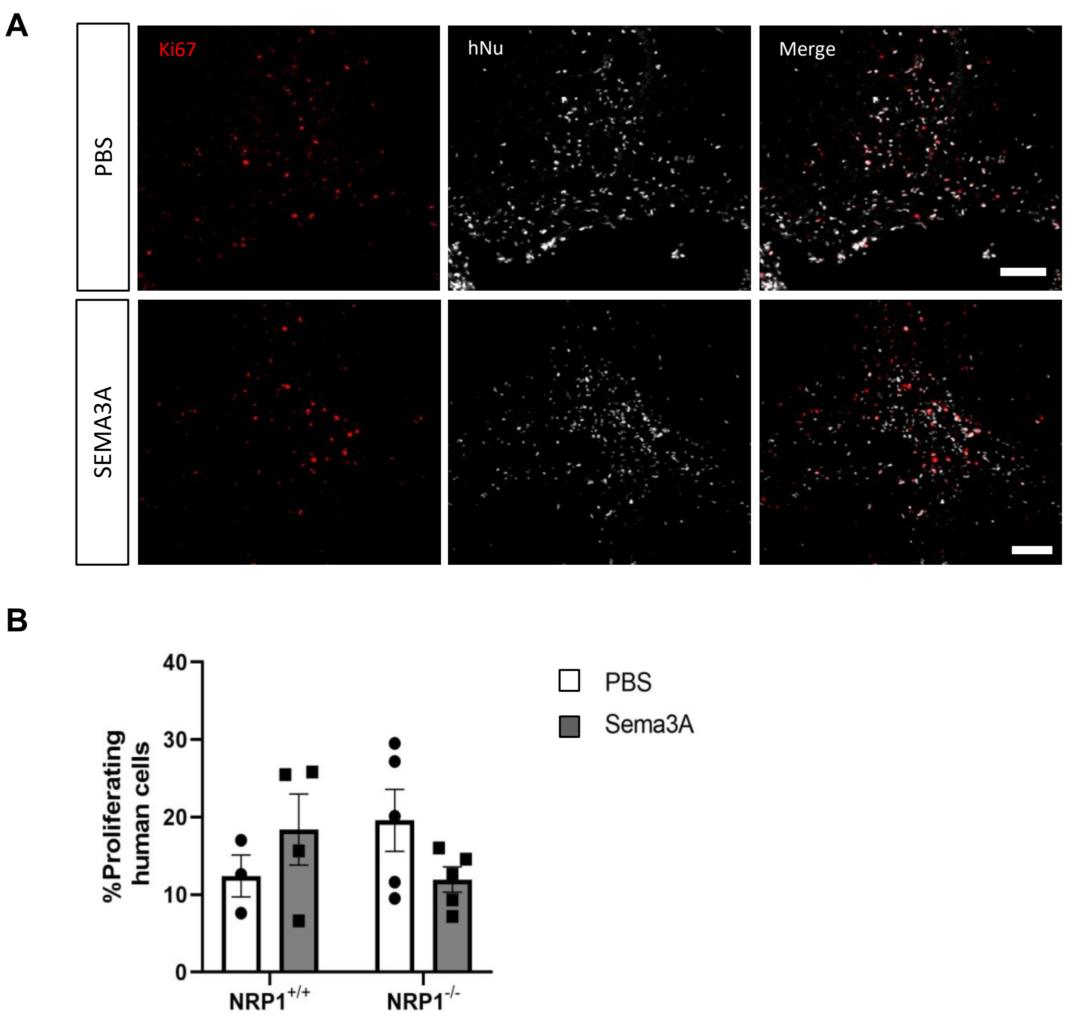

Supplementary Figure 5: NRP1$^{+/+}$ and NRP1$^{-/-}$ human cell proliferation in the lesion is not affected by SEMA3A. (A) Representative images of Ki67 (red) and human nuclear marker (hNu, white) immunofluorescent staining at the lesion site of PBS or SEMA3A-loaded lesions. Here, showing NRP1$^{-/-}$ cells. Scale bars = 100μm. (B) The percentage of proliferating human cells determined by the number of Ki67+hNu+ human nuclei. No significant difference in proliferation was observed between cell genotypes (p=0.3518 and p=0.3656) or condition (p=0.5036 and p=0.2193). Two-way ANOVA with Sidak's test. NRP1$^{+/+}$ n=3 (PBS) n=4 (SEMA3A), NRP1$^{-/-}$ n=5 both. Each point represents 1 mouse and is the average quantification of 3 sections/mouse. Graph plots: means ± SEM.

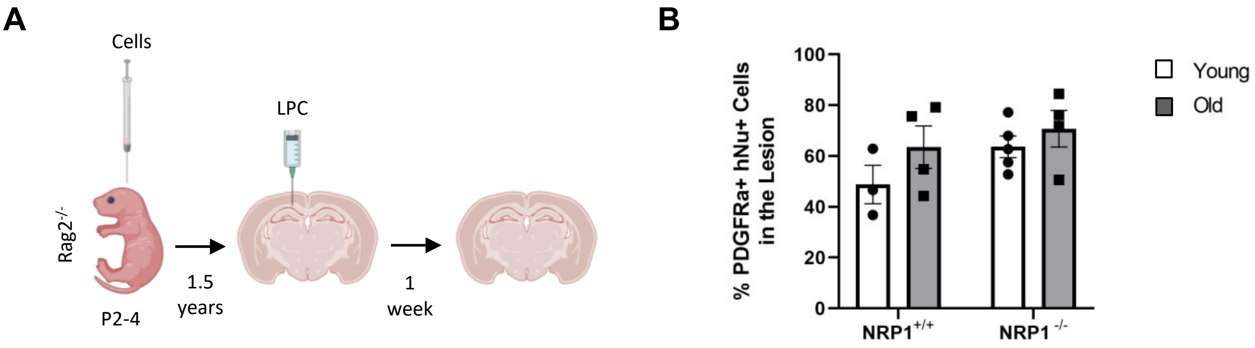

Supplementary Figure 6: Following demyelination, hOPCs migrate effectively in both young and aged mice. (A) Experimental outline. P2-4 Rag2-/- pups were injected with NRP1$^{+/+}$ or NRP1$^{-/-}$ cells. 1.5 years later, adult mice received an LPC lesion. Analysis was performed 1 week post lesion. (B) The percentage of NRP1$^{+/+}$ or NRP1$^{-/-}$ PDGFRα+ hOPCs in the lesion is similar between young and old mice. Two-way ANOVA with Sidak's test. Young NRP1$^{+/+}$ n=3, Young NRP1$^{-/-}$ n=5, Old NRP1$^{+/+}$ and NRP1$^{-/-}$ n=4. Each point represents 1 mouse, and is the average quantification of 3 sections/mouse. Graph plots: means ± SEM.

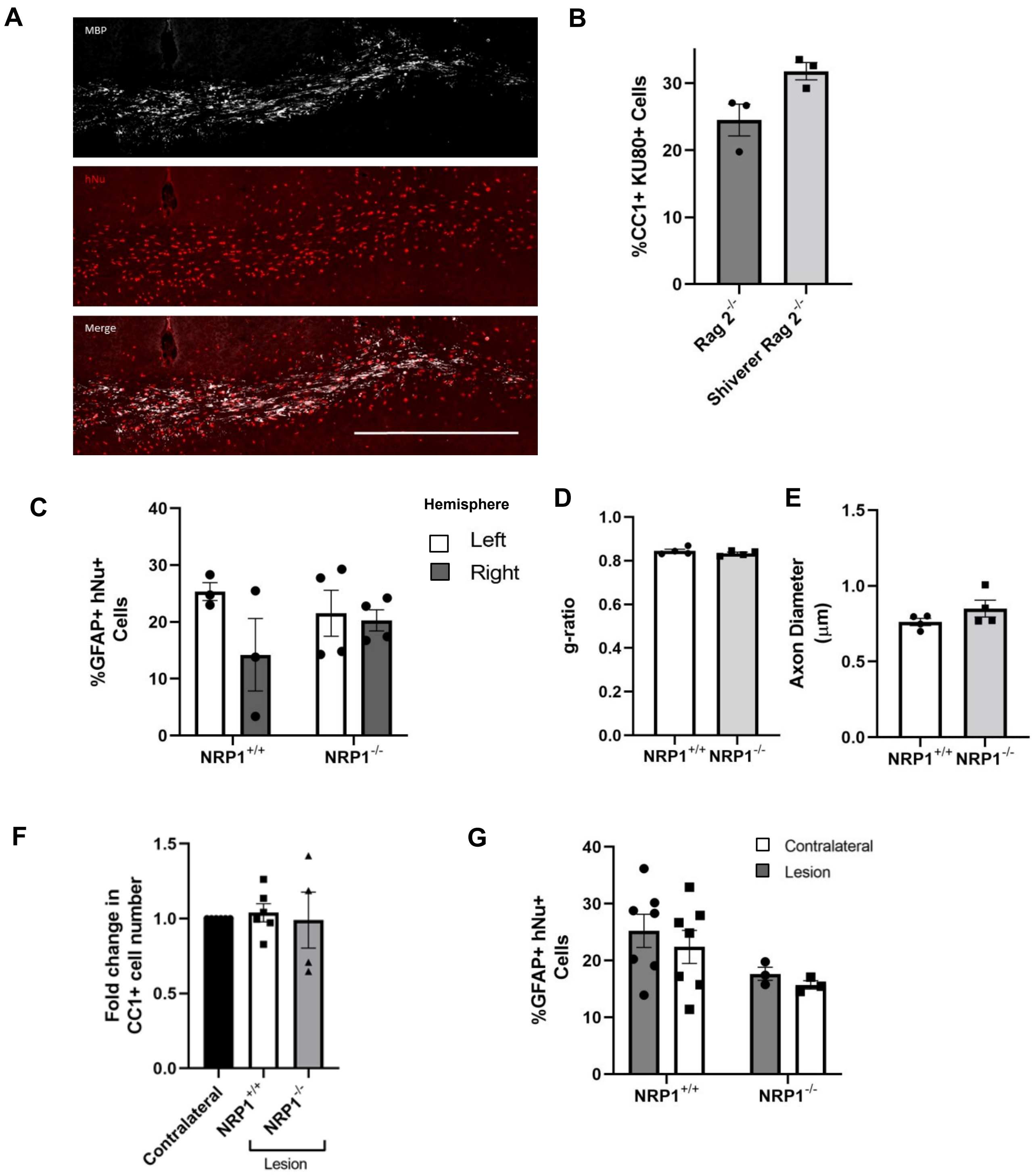
A
MBP
hNu
Merge
B
%CC1+ KU80+ Cells
0
10
20
30
Rag 2-/-
Shiverer Rag 2-/-
C
%GFAP+ hNu+ Cells
0
10
20
30
40
Hemisphere
Left
Right
NRP1+/+
NRP1-/-
D
g-ratio
0.0
0.2
0.4
0.6
0.8
1.0
NRP1+/+
NRP1-/-
E
Axon Diameter (μm)
0.0
0.5
1.0
1.5
NRP1+/+
NRP1-/-
F
Fold change in CC1+ cell number
0.0
0.5
1.0
1.5
Contralateral
NRP1+/+
NRP1-/-
Lesion
G
%GFAP+ hNu+ Cells
0
10
20
30
40
Contralateral
Lesion
NRP1+/+
NRP1-/-

Supplementary Figure 7: Analysis of human myelin in *Shiverer:Rag2$^{-/-}$* mice (A) MBP+ human myelin in the corpus callosum 6 weeks post transplant. Scale bar = 500µm. (B) NRP1$^{+/+}$ and NRP1$^{-/-}$ cells generate equal numbers of GFAP+ astrocytes in uninjured *Shi/Shi:Rag2$^{-/-}$* mice, that are evenly distributed in both hemispheres. n=3 NRP1$^{+/+}$ n=4 NRP1$^{-/-}$ for each condition, two-tailed unpaired t-test, p=0.1663 and p= 0.789. (C) Average g-ratios showing no difference in myelin thickness between NRP1$^{+/+}$ and NRP1$^{-/-}$ transplants. n=4 for each condition, unpaired two-tailed t-test, p=0.3586. (D) The average diameter of myelinated axons was not different between NRP1$^{+/+}$ and NRP1$^{-/-}$ transplants. n=4 for each condition, unpaired two-tailed t-test, p=0.1944. (E) 3 weeks post lesion, the number of CC1-positive human oligodendrocytes was equal between the uninjured contralateral and the lesion sites when either NRP1$^{+/+}$ (p=0.9399) or NRP1$^{-/-}$ (p=0.9969) cells were transplanted. n=6/4 respectively, one-way ANOVA, Tukey test. (F) NRP1$^{+/+}$ and NRP1$^{-/-}$ GFAP+ astrocyte numbers were similar between the lesion and the unlesioned contralateral side. n=7/3 respectively for each condition, two-tailed unpaired t-test, p=0.5034 and p=0.2269. For B,E,F: each point represents 1 mouse, and is the average quantification of 3 sections/mouse. For C,D: each point represents 1 mouse, and is the average quantification of ~100 axons per mouse. All graphs plot means ± SEM.

A

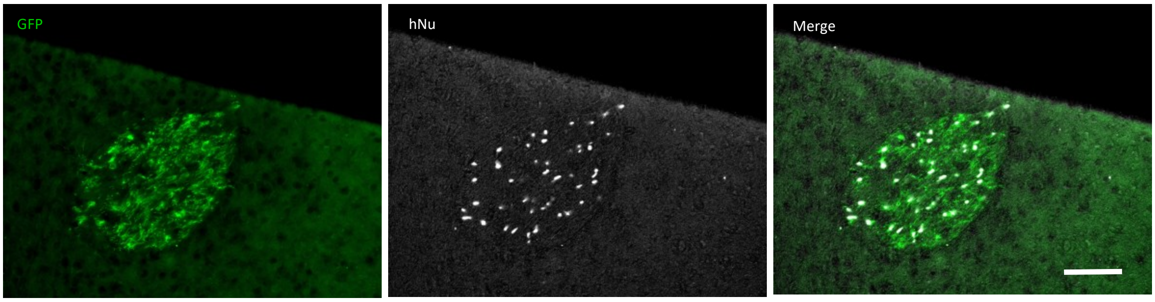

B

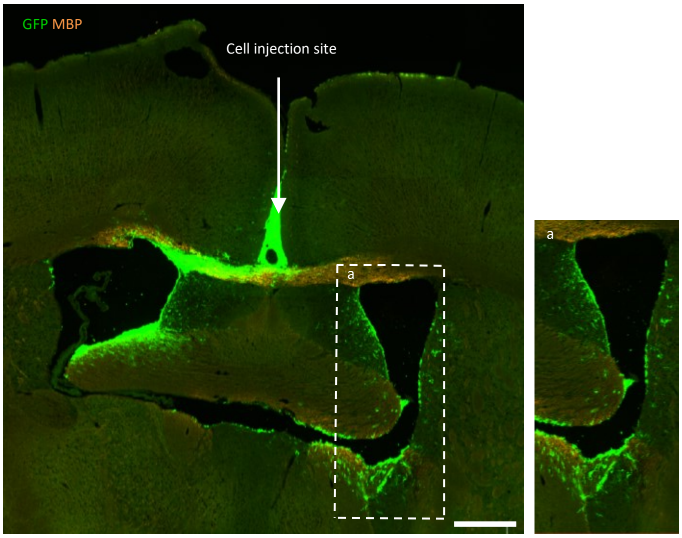

Supplementary Figure 8: Transplanted cell distribution in the brain. (A) Following long-term transplantation into the parenchyma, cells were even found in the anterior commissure. Scale bar 100µm (B) hOPCs injected into the cerebral spinal fluid (CSF) were highly migratory and found far away from the injection site. Scale bar 500µm. Representative images.

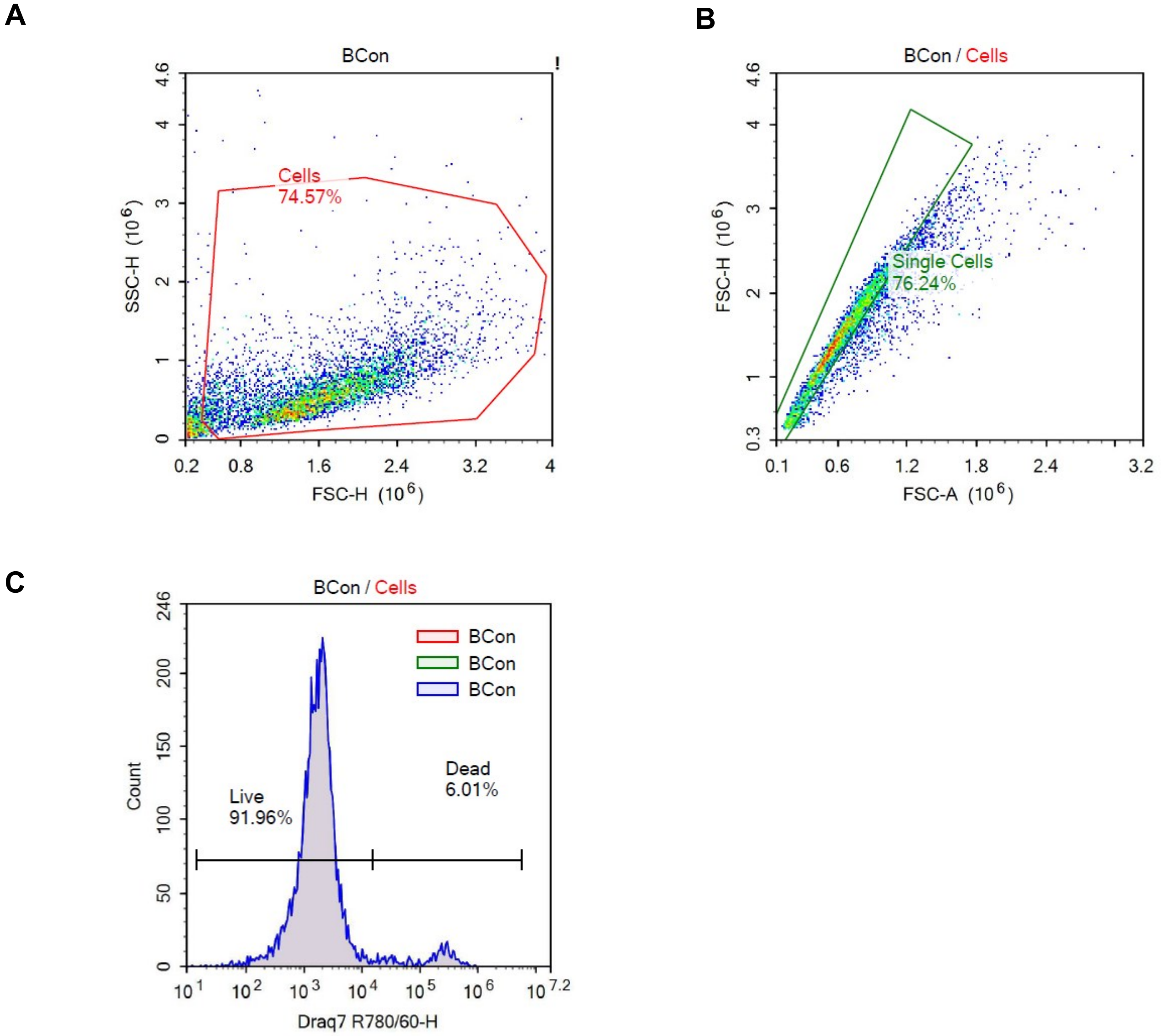

Supplementary Figure 9: Gating strategy for live/dead flow cytometry. Examples from sample B control (BCon). (A) Forward and side scatter gating to select all cells. (B) Forward scatter gating to select single cells. (C) Draq7 histogram determining live and dead cells.

Supplemental Table 1: OPC differential expression.

Gene	p_val	avg_log2FC	pct.1	pct.2	p_val_adj
CASK	9.95E-27	-0.2543	0.806	0.894	2.97E-22
MDK	5.05E-21	-0.3059	0.242	0.429	1.51E-16
USP34	2.16E-18	0.25446	0.514	0.327	6.44E-14
ODC1	2.05E-17	-0.26017	0.516	0.658	6.12E-13
HMGN2	2.13E-12	-0.25683	0.607	0.726	6.34E-08

Supplemental Table 2: Oligodendroglia differential expression.

Gene	p_val	avg_log2FC	pct.1	pct.2	p_val_adj
CASK	1.69E-84	-0.31517	0.688	0.817	5.04E-80
MDK	6.38E-76	-0.38219	0.333	0.552	1.90E-71
MT3	1.84E-75	-0.41041	0.282	0.476	5.48E-71
USP34	1.31E-57	0.276567	0.522	0.314	3.90E-53
ODC1	3.88E-53	-0.28373	0.455	0.608	1.16E-48
HOPX	7.81E-44	-0.288	0.47	0.577	2.33E-39
PTGDS	1.44E-38	0.310825	0.322	0.169	4.30E-34

Supplemental Table 3: Astrocytes differential expression.

Gene	p_val	avg_log2FC	pct.1	pct.2	p_val_adj
MT3	0	-0.56292493	0.553	0.829	0
NEAT1	6.11E-186	-0.43388126	0.496	0.728	1.82E-181
OTX2-AS1	4.04E-150	0.343190753	0.384	0.141	1.20E-145
MDK	1.24E-135	-0.33201636	0.627	0.804	3.69E-131
S100A13	8.81E-113	-0.30389208	0.193	0.39	2.63E-108
USP34	2.41E-103	0.281297101	0.514	0.317	7.18E-99
CASK	5.74E-98	-0.25934495	0.616	0.75	1.71E-93
NTRK2	1.66E-93	0.278744217	0.358	0.172	4.95E-89
DCLK1	2.48E-93	0.268416088	0.77	0.619	7.39E-89
PTGDS	2.94E-90	0.304468934	0.306	0.131	8.75E-86
LGALS1	2.02E-74	-0.27985847	0.354	0.513	6.03E-70
EDNRB	1.77E-68	-0.28779725	0.634	0.758	5.28E-64

Supplemental Table 4: All cells differential expression.

Gene	p_val	avg_log2FC	pct.1	pct.2	p_val_adj
MT3	0	-0.46542596	0.404	0.615	0
OTX2-AS1	3.19E-202	0.278029875	0.264	0.091	9.52E-198
CASK	1.12E-198	-0.28886145	0.632	0.768	3.33E-194
MDK	1.20E-188	-0.32124218	0.468	0.636	3.59E-184
USP34	3.39E-188	0.290112295	0.512	0.312	1.01E-183
PTGDS	1.72E-134	0.278377612	0.273	0.124	5.12E-130
NEAT1	1.32E-129	-0.291891	0.408	0.551	3.93E-125

Supplemental Table 5: Primary antibody information

Antibody	Source	Identifier	Dilution	Application
Rat monoclonal anti-MBP	Serotec	MCA409S RRID:AB_325004	1:250	IF
Rabbit polyclonal anti-Olig2	Millipore	AB9610 RRID:AB_570666	1:400	IF
Goat polyclonal anti-Olig2	R&D Systems	AF2418 RRID:AB_2157554	1:400	IF
Mouse monoclonal anti-Nuclei Antibody, clone 235-1	Millipore	MAB1281 RRID:AB_94090	1:400	IF
Mouse monoclonal anti-Oligodendrocyte Marker O4	R&D Systems	MAB1326 RRID:AB_357617	1:400	IF
Rabbit polyclonal anti-Ki67	Abcam	ab15580 RRID:AB_443209	1:400	IF
Rabbit monoclonal anti-IBA1	Abcam	ab178846 RRID:AB_2636859	1:400	IF
Rabbit monoclonal anti-PDGFRa	Cell signalling	3174 RRID:AB_2162345	Tissue 1:400 cells 1:200	IF
Rabbit monoclonal anti-KU80	Cell signalling	2180 RRID:AB_2218736	1:400	IF
Mouse monoclonal anti-APC (CC1)	Abcam	ab16794 RRID:AB_443473	1:100	IF
Chicken anti-GFAP	Cambridge Bioscience	829401	1:500	IF
Mouse monoclonal anti-beta actin	Sigma	A2228 RRID:AB_476697	1:1000	WB
Rabbit monoclonal anti-NRP1	Abcam	ab81321 RRID:AB_1640739	1:1000	WB
Sheep polyclonal anti-NRP1	R&D Systems	AF3870 RRID:AB_884367	1:200	WB
