# Supplementary material for: CRISPR-edited human ES-derived oligodendrocyte progenitor cells improve remyelination in rodents
Source: Nat Commun. 2024 Oct 9;15:8570. doi: 10.1038/s41467-024-52444-w (PMC11464782; doi:10.1038/s41467-024-52444-w)
Supplement: Supplementary file 3 — Description of Additional Supplementary Files [file 41467_2024_52444_MOESM3_ESM.pdf]

Description of Additional Supplementary Files

Supplementary Video 1: Human oligodendrocyte myelination of mouse axons. A z-stack showing Human GFP+ (green) oligodendrocytes generating MBP (red) 10 weeks after transplantation into Shi/Shi:Rag2-/- mice. Hoechst (blue). Corresponding still shown in Supplementary Figure 1 E.

Supplementary Data 1: Copy Number Variation The tab “all CNVs” contains the location, size and copy number value of all the CNVs detected with the SNP array in the NRP1+/+ and NRP1-/- cells. In green are highlighted the CNVs that are identical between the two samples. The second tab contains the CNVs found in the NRP1-/- cells but absent in the NRP1+/+

Supplementary Data 2: CAS9 off-target predicted sequences
