# Supplementary material for: CRISPR-edited human ES-derived oligodendrocyte progenitor cells improve remyelination in rodents
Source: Nat Commun. 2024 Oct 9;15:8570. doi: 10.1038/s41467-024-52444-w (PMC11464782; doi:10.1038/s41467-024-52444-w)
Supplement: Supplementary file 7 — Reporting Summary [file 41467_2024_52444_MOESM7_ESM.pdf]

nature portfolio

Corresponding author(s): Laura Wagstaff and Anna Williams

Last updated by author(s): 11-07-2024

Reporting Summary

Nature Portfolio wishes to improve the reproducibility of the work that we publish. This form provides structure for consistency and transparency in reporting. For further information on Nature Portfolio policies, see our Editorial Policies and the Editorial Policy Checklist.

Statistics

For all statistical analyses, confirm that the following items are present in the figure legend, table legend, main text, or Methods section.

n/a	Confirmed	
☐	☒	The exact sample size (n) for each experimental group/condition, given as a discrete number and unit of measurement
☐	☒	A statement on whether measurements were taken from distinct samples or whether the same sample was measured repeatedly
☐	☒	The statistical test(s) used AND whether they are one- or two-sided<br>*Only common tests should be described solely by name; describe more complex techniques in the Methods section.*
☐	☒	A description of all covariates tested
☐	☒	A description of any assumptions or corrections, such as tests of normality and adjustment for multiple comparisons
☐	☒	A full description of the statistical parameters including central tendency (e.g. means) or other basic estimates (e.g. regression coefficient) AND variation (e.g. standard deviation) or associated estimates of uncertainty (e.g. confidence intervals)
☐	☒	For null hypothesis testing, the test statistic (e.g. F, t, r) with confidence intervals, effect sizes, degrees of freedom and P value noted<br>*Give P values as exact values whenever suitable.*
☒	☐	For Bayesian analysis, information on the choice of priors and Markov chain Monte Carlo settings
☒	☐	For hierarchical and complex designs, identification of the appropriate level for tests and full reporting of outcomes
☒	☐	Estimates of effect sizes (e.g. Cohen's d, Pearson's r), indicating how they were calculated

Our web collection on statistics for biologists contains articles on many of the points above.

Software and code

Policy information about availability of computer code

Data collection	Immunofluorescence was imaged on a wide field Zeiss observer using Zeiss Zen (blue edition) and a Leica TCS SP8 confocal. Single cell RNA sequencing libraries were sequenced using a NovaSeq 6000 sequencingsystem (PE150 (HiSeq), Illumina). SNP array experiments were performed using the CytoSNP 850K BeadChip from Illumina.
Data analysis	Manual cell counts were performed using Fuji/ImageJ (v1.54) or Qupath software (v0.3.1). MBP percentage area was analysed using Fuji/ ImageJ. Axons were counted in Fiji/ImageJ. g-ratios were measured using Qupath. Data handling was managed on Microsoft Excel. Statistical analysis was performed using Graphpad Prism (8.3.0). Single cell RNA sequences were aligned to the reference genome, feature counting and cell calling were performed following the 10x Genomics CellRanger (v.7.0.0) pipelinewith the human reference genome provided by 10x-refdata-gex-GRCh38-2020-A. The downstream analysis was performed in Rv4.2.1, with the QC performed with scater, using the outliers from isOutlier function for the library size filtering and a threshold of 6%of mitochondrial genes. The normalisation, feature variance estimation and dimensional reduction were performed with scran, with logNormCounts(), modelGeneVar() andrunPCA()/runTSNE() respectively. Clustering was performed with Seurat's implementation of the Louvain clustering. The clusters were annotated using canonical markers and lists of differentially expressed markers between clusters, obtained with wilcoxauc() from the immunogenomics /presto package. A Differential Expression analysis between NRP1-/- and NRP1+/+ cells was performed on oligodendroglia, OPCs and astrocytes with MAST using Seurat's FindMarkers() wrapper with the default thresholds of 0.25 logFC and 0.1 pct cells expressing the gene. SNP array experiments were analysed in GenomeStudio 2.0 with the plug-in cnvPartition 3.2.0.

For manuscripts utilizing custom algorithms or software that are central to the research but not yet described in published literature, software must be made available to editors and reviewers. We strongly encourage code deposition in a community repository (e.g. GitHub). See the Nature Portfolio guidelines for submitting code & software for further information.

Data

Policy information about availability of data

All manuscripts must include a data availability statement. This statement should provide the following information, where applicable:

- Accession codes, unique identifiers, or web links for publicly available datasets
- A description of any restrictions on data availability
- For clinical datasets or third party data, please ensure that the statement adheres to our policy

The data that support the findings in this study are available as unrestricted source data files. Our RNAseq data is open access already and available at https://www.ncbi.nlm.nih.gov/geo/query/acc.cgi?acc=GSE241451. Accession number: GSE241451.

Research involving human participants, their data, or biological material

Policy information about studies with human participants or human data. See also policy information about sex, gender (identity/presentation), and sexual orientation and race, ethnicity and racism.

Reporting on sex and gender	N/A
Reporting on race, ethnicity, or other socially relevant groupings	N/A
Population characteristics	N/A
Recruitment	N/A
Ethics oversight	N/A

Note that full information on the approval of the study protocol must also be provided in the manuscript.

Field-specific reporting

Please select the one below that is the best fit for your research. If you are not sure, read the appropriate sections before making your selection.

☒ Life sciences ☐ Behavioural & social sciences ☐ Ecological, evolutionary & environmental sciences

For a reference copy of the document with all sections, see nature.com/documents/nr-reporting-summary-flat.pdf

Life sciences study design

All studies must disclose on these points even when the disclosure is negative.

Sample size	This was a hypothesis driven study. Sample sizes were based on our previous mouse studies (Boyd et al., 2013, Piaton et al., 2011, Williams et al., 2007), estimating similar effect sizes.
Data exclusions	No data was excluded from the anaylsis
Replication	Technical and biological repeats are described in the text/methods/legends.
Randomization	Mice and cell samples were randomly assigned to each experimental group
Blinding	Experimentor was blinded during all data collection and analysis

Reporting for specific materials, systems and methods

We require information from authors about some types of materials, experimental systems and methods used in many studies. Here, indicate whether each material, system or method listed is relevant to your study. If you are not sure if a list item applies to your research, read the appropriate section before selecting a response.

Materials & experimental systems

n/a	Involved in the study
☐	☒ Antibodies
☐	☒ Eukaryotic cell lines
☐	☐ Palaeontology and archaeology
☐	☒ Animals and other organisms
☐	☐ Clinical data
☐	☐ Dual use research of concern
☐	☐ Plants

Methods

n/a	Involved in the study
☐	☐ ChIP-seq
☐	☒ Flow cytometry
☐	☐ MRI-based neuroimaging

Antibodies

Antibodies used

Rat monoclonal anti-MBP Serotec MCA409S RRID:AB_325004 (1:250) Rabbit polyclonal anti-Olig2 Millipore AB9610 RRID:AB_570666 (1:400) Goat polyclonal anti-Olig2 R&DSystems AF2418 RRID:AB_2157554 (1:400) Mouse monoclonal anti-NucleiAntibody, clone235-1 Millipore MAB1281 RRID:AB_94090 (1:400) Mouse monoclonal anti-Oligodendrocyte Marker O4 R&DSystems MAB1326 RRID:AB_357617 (1:400) Rabbit polyclonal anti-Ki67 Abcam ab15580 RRID:AB_443209 (1:400) Rabbit monoclonal anti-IBA1 Abcam ab178846 RRID:AB_2636859 (1:400) Rabbit monoclonal anti-PDGFRa Cellsignalling 3174 RRID:AB_2162345 (Tissue1:400, cells1:200) Mouse monoclonal anti-beta actin Sigma A2228 RRID:AB_476697 (1:1000) Rabbit monoclonal anti-NRP1 Abcam ab81321 RRID:AB_1640739 (1:1000) Sheep polyclonal anti-NRP1 R&DSystems AF3870 RRID:AB_884367 (1:200) Rabbit monoclonal anti-KU80 Cell signalling 2180 RRID:AB_2218736 (1:400) Mouse monoclonal anti-APC (CC1) Abcam ab16794 RRID:AB_443473 (1:100) Chicken anti-GFAP Cambridge Bioscience 829401 (1:500)

Validation

Validation of the antiboides used were stated on the suppliers websites. We tested NRP1 antibodies on whole mouse brain lysate and HUVECs cells. MBP, Olig2, O4, PDGFRa and Ki67 produced signal consistent with ours and others previous findings - Miron et al. Nat Neurosci, (2013), Quick et al. Acta Neuropathol, (2022), Livesey et al. Stem Cells, (2016), McNamara et al. Nature (2022) Anti-NucleiAntibody, clone235-1 was similar to that reported by Windrem et al. Cell Stem Cell (2008). KU80 was similar to that reported by Allard et al. Regen Med (2015).

Eukaryotic cell lines

Policy information about cell lines and Sex and Gender in Research

Cell line source(s)

RC17 GMP grade, female, human embroynic cell line was used - De Sousa et al. Stem Cell Research, (2016)

Authentication

n/a

Mycoplasma contamination

nil

Commonly misidentified lines
(See ICLAC register)

n/a

Palaeontology and Archaeology

Specimen provenance

Provide provenance information for specimens and describe permits that were obtained for the work (including the name of the issuing authority, the date of issue, and any identifying information). Permits should encompass collection and, where applicable, export.

Specimen deposition

Indicate where the specimens have been deposited to permit free access by other researchers.

Dating methods

If new dates are provided, describe how they were obtained (e.g. collection, storage, sample pretreatment and measurement), where they were obtained (i.e. lab name), the calibration program and the protocol for quality assurance OR state that no new dates are provided.

☐ Tick this box to confirm that the raw and calibrated dates are available in the paper or in Supplementary Information.

Ethics oversight

Identify the organization(s) that approved or provided guidance on the study protocol, OR state that no ethical approval or guidance was required and explain why not.

Note that full information on the approval of the study protocol must also be provided in the manuscript.

Animals and other research organisms

Policy information about studies involving animals; ARRIVE guidelines recommended for reporting animal research, and Sex and Gender in Research

Laboratory animals	Rag2-/- P2-4, 8 weeks, 1.5 years old. Shi/Shi:Rag 2-/- P2-4, 6 and 10 weeks.
Wild animals	n/a
Reporting on sex	Mice of both sexes were used
Field-collected samples	n/a
Ethics oversight	Experiments were performed under a UK Home Office Licence granted to Anna Williams.

Note that full information on the approval of the study protocol must also be provided in the manuscript.

Clinical data

Policy information about clinical studies

All manuscripts should comply with the ICMJE guidelines for publication of clinical research and a completed CONSORT checklist must be included with all submissions.

Clinical trial registration	*Provide the trial registration number from ClinicalTrials.gov or an equivalent agency.*
Study protocol	*Note where the full trial protocol can be accessed OR if not available, explain why.*
Data collection	*Describe the settings and locales of data collection, noting the time periods of recruitment and data collection.*
Outcomes	*Describe how you pre-defined primary and secondary outcome measures and how you assessed these measures.*

Dual use research of concern

Policy information about dual use research of concern

Hazards

Could the accidental, deliberate or reckless misuse of agents or technologies generated in the work, or the application of information presented in the manuscript, pose a threat to:

No	Yes	
☒	☐	Public health
☒	☐	National security
☒	☐	Crops and/or livestock
☒	☐	Ecosystems
☒	☐	Any other significant area

Experiments of concern

Does the work involve any of these experiments of concern:

No	Yes	
☒	☐	Demonstrate how to render a vaccine ineffective
☒	☐	Confer resistance to therapeutically useful antibiotics or antiviral agents
☒	☐	Enhance the virulence of a pathogen or render a nonpathogen virulent
☒	☐	Increase transmissibility of a pathogen
☒	☐	Alter the host range of a pathogen
☒	☐	Enable evasion of diagnostic/detection modalities
☒	☐	Enable the weaponization of a biological agent or toxin
☒	☐	Any other potentially harmful combination of experiments and agents

Plants

Seed stocks	*Report on the source of all seed stocks or other plant material used. If applicable, state the seed stock centre and catalogue number. If plant specimens were collected from the field, describe the collection location, date and sampling procedures.*
Novel plant genotypes	*Describe the methods by which all novel plant genotypes were produced. This includes those generated by transgenic approaches, gene editing, chemical/radiation-based mutagenesis and hybridization. For transgenic lines, describe the transformation method, the number of independent lines analyzed and the generation upon which experiments were performed. For gene-edited lines, describe the editor used, the endogenous sequence targeted for editing, the targeting guide RNA sequence (if applicable) and how the editor was applied.*
Authentication	*Describe any authentication procedures for each seed stock used or novel genotype generated. Describe any experiments used to assess the effect of a mutation and, where applicable, how potential secondary effects (e.g. second site T-DNA insertions, mosiacism, off-target gene editing) were examined.*

ChIP-seq

Data deposition

☐ Confirm that both raw and final processed data have been deposited in a public database such as GEO.

☐ Confirm that you have deposited or provided access to graph files (e.g. BED files) for the called peaks.

Data access links<br>*May remain private before publication.*	*For "Initial submission" or "Revised version" documents, provide reviewer access links. For your "Final submission" document, provide a link to the deposited data.*
Files in database submission	*Provide a list of all files available in the database submission.*
Genome browser session<br>(e.g. UCSC)	*Provide a link to an anonymized genome browser session for "Initial submission" and "Revised version" documents only, to enable peer review. Write "no longer applicable" for "Final submission" documents.*

Methodology

Replicates	*Describe the experimental replicates, specifying number, type and replicate agreement.*
Sequencing depth	*Describe the sequencing depth for each experiment, providing the total number of reads, uniquely mapped reads, length of reads and whether they were paired- or single-end.*
Antibodies	*Describe the antibodies used for the ChIP-seq experiments; as applicable, provide supplier name, catalog number, clone name, and lot number.*
Peak calling parameters	*Specify the command line program and parameters used for read mapping and peak calling, including the ChIP, control and index files used.*
Data quality	*Describe the methods used to ensure data quality in full detail, including how many peaks are at FDR 5% and above 5-fold enrichment.*
Software	*Describe the software used to collect and analyze the ChIP-seq data. For custom code that has been deposited into a community repository, provide accession details.*

Flow Cytometry

Plots

Confirm that:

☒ The axis labels state the marker and fluorochrome used (e.g. CD4-FITC).

☒ The axis scales are clearly visible. Include numbers along axes only for bottom left plot of group (a 'group' is an analysis of identical markers).

☒ All plots are contour plots with outliers or pseudocolor plots.

☒ A numerical value for number of cells or percentage (with statistics) is provided.

Methodology

Sample preparation	hOPCs were cultured in differentiation media with or without SEMA3A (5µg/ml) for 72hours. They were then lifted with accutase and resuspended in PBS with approximately 50000 cells per 100ul. Samples were stained with DRAQ7™ (1:1000, Abcam) to detect dead and membrane-compromised cells.
Instrument	Samples were analysed using The NovoCyte Advanteon flow cytometer (Agilent Technologies, Inc. 2021)

Software	NovoExpress 1.5.0 software (Agilent Technologies, Inc. 2021) determined live/dead cell populations
Cell population abundance	N/A
Gating strategy	Cells and single cells were identified from forward and side scatter plots. Single cells were further gated on live/dead staining

☒ Tick this box to confirm that a figure exemplifying the gating strategy is provided in the Supplementary Information.

Magnetic resonance imaging

Experimental design

Design type	*Indicate task or resting state; event-related or block design.*
Design specifications	*Specify the number of blocks, trials or experimental units per session and/or subject, and specify the length of each trial or block (if trials are blocked) and interval between trials.*
Behavioral performance measures	*State number and/or type of variables recorded (e.g. correct button press, response time) and what statistics were used to establish that the subjects were performing the task as expected (e.g. mean, range, and/or standard deviation across subjects).*

Acquisition

Imaging type(s)	*Specify: functional, structural, diffusion, perfusion.*
Field strength	*Specify in Tesla*
Sequence & imaging parameters	*Specify the pulse sequence type (gradient echo, spin echo, etc.), imaging type (EPI, spiral, etc.), field of view, matrix size, slice thickness, orientation and TE/TR/flip angle.*
Area of acquisition	*State whether a whole brain scan was used OR define the area of acquisition, describing how the region was determined.*

Diffusion MRI ☐ Used ☐ Not used

Preprocessing

Preprocessing software	*Provide detail on software version and revision number and on specific parameters (model/functions, brain extraction, segmentation, smoothing kernel size, etc.).*
Normalization	*If data were normalized/standardized, describe the approach(es): specify linear or non-linear and define image types used for transformation OR indicate that data were not normalized and explain rationale for lack of normalization.*
Normalization template	*Describe the template used for normalization/transformation, specifying subject space or group standardized space (e.g. original Talairach, MNI305, ICBM152) OR indicate that the data were not normalized.*
Noise and artifact removal	*Describe your procedure(s) for artifact and structured noise removal, specifying motion parameters, tissue signals and physiological signals (heart rate, respiration).*
Volume censoring	*Define your software and/or method and criteria for volume censoring, and state the extent of such censoring.*

Statistical modeling & inference

Model type and settings	*Specify type (mass univariate, multivariate, RSA, predictive, etc.) and describe essential details of the model at the first and second levels (e.g. fixed, random or mixed effects; drift or auto-correlation).*
Effect(s) tested	*Define precise effect in terms of the task or stimulus conditions instead of psychological concepts and indicate whether ANOVA or factorial designs were used.*

Specify type of analysis: ☐ Whole brain ☐ ROI-based ☐ Both

Statistic type for inference (See Eklund et al. 2016)	*Specify voxel-wise or cluster-wise and report all relevant parameters for cluster-wise methods.*
Correction	*Describe the type of correction and how it is obtained for multiple comparisons (e.g. FWE, FDR, permutation or Monte Carlo).*

Models & analysis

n/a	Involved in the study
☐	☐ Functional and/or effective connectivity
☐	☐ Graph analysis
☐	☐ Multivariate modeling or predictive analysis

Functional and/or effective connectivity	*Report the measures of dependence used and the model details (e.g. Pearson correlation, partial correlation, mutual information).*
Graph analysis	*Report the dependent variable and connectivity measure, specifying weighted graph or binarized graph, subject- or group-level, and the global and/or node summaries used (e.g. clustering coefficient, efficiency, etc.).*
Multivariate modeling and predictive analysis	*Specify independent variables, features extraction and dimension reduction, model, training and evaluation metrics.*
