# Supplementary material for: CRISPR-edited human ES-derived oligodendrocyte progenitor cells improve remyelination in rodents
Source: Nat Commun. 2024 Oct 9;15:8570. doi: 10.1038/s41467-024-52444-w (PMC11464782; doi:10.1038/s41467-024-52444-w)
Supplement: Supplementary file 2 — Peer Review File [file 41467_2024_52444_MOESM2_ESM.pdf]

nature portfolio

Peer Review File

CRISPR-edited human ES-derived oligodendrocyte progenitor cells improve remyelination in rodents

Open Access This file is licensed under a Creative Commons Attribution 4.0 International License, which permits use, sharing, adaptation, distribution and reproduction in any medium or format, as long as you give appropriate credit to the original author(s) and the source, provide a link to the Creative Commons license, and indicate if changes were made. In the cases where the authors are anonymous, such as is the case for the reports of anonymous peer reviewers, author attribution should be to 'Anonymous Referee' followed by a clear attribution to the source work. The images or other third party material in this file are included in the article's Creative Commons license, unless indicated otherwise in a credit line to the material. If material is not included in the article's Creative Commons license and your intended use is not permitted by statutory regulation or exceeds the permitted use, you will need to obtain permission directly from the copyright holder. To view a copy of this license, visit http://creativecommons.org/licenses/by/4.0/.

REVIEWER COMMENTS

Reviewer #1 (Remarks to the Author):

In this manuscript, Wagstaff et al. generated human OPCs from human embryonic stem cells and introduced them to young and aged mice that undergo lysolecithin-induced focal demyelination. The experiments demonstrate that hOPCs injected into the mouse corpus callosum are capable of surviving and migrating along the corpus callosum into areas with demyelination. My main concern with the manuscript, given its primary focus on the therapeutic potential of transplanting hOPCs for remyelination, lies in its relevance to human demyelinating disease. As it stands, the data convincingly demonstrate that deleting NRP1 from OPCs blocks their responsiveness to Sema3A, but not that Sema3A contributes to inefficient remyelination in demyelinating disease. Major concerns are described below:

Major:

- Overall, the data presented here indicate that the artificial addition of Sema3A is required for the knockout of NRP1 to influence OPC migration into LPC lesions in mice. While it may be the case that chronic lesions in humans contain higher levels of Sema3A than rodent LPC injections, it is not clear from the data presented in this paper whether Sema3A is a critical inhibitor of remyelination in that context.
- The authors also mention that ageing contributes to reduced recruitment of OPCs to lesions, but their data in Figure 3E, F, and S4B show no difference in recruitment between young and old mice, which suggests that LPC-induced demyelination in mice may not be a suitable model for studying ageing-related factors that reduce OPC recruitment. Perhaps a model more similar to human demyelination, such as EAE, could better recapitulate some of the factors limiting OPC recruitment to lesions in human demyelinating disease that the authors hope to alleviate with the knockout of NRP1.
- The rationale for injecting hOPCs perinatally is unclear from a therapeutic perspective.
- What are the hESC-derived cells that don't express PDGFRa or Olig2? Some attempt to characterize the identity of all the injected cells would be useful for interpretation.
- What is the % of mature oligodendrocytes that arose from injected cells? Is this % more or less in the NRP1-/- cells, and does that lead to an overall higher number of mature OLs in the corpus callosum?

Minor:

- In Figure S1E, why is there little overlap between GFP and MBP? Seems like the majority of GFP+ sheaths are not MBP+.
- More zoomed out views of the corpus callosum (similar to Figure 3B) should be included in Figure S2.
- Figure 3I should include example images from a Sema3A lesion injected with NRP1-/- cells.

Reviewer #2 (Remarks to the Author):

The manuscript by Wagstaff and colleagues has investigated the utility of genetic modification of human OPCs (hOPCs) prior to transplantation in overcoming the non-permissive environment of chronic active lesions to achieve remyelination in multiple sclerosis. This is an extremely important issue given that clinical trials of drugs that promote differentiation of endogenous OPCs have not shown major benefits in patients with MS, likely due to paucity of OPCs in chronic lesions, age-induced lack of responsiveness to pro-differentiating stimuli, as well as limited access of these drugs to the lesions. These issues, in addition to the fact that low oligodendroglial numbers are associated with MS progression, strongly indicate that repopulating MS brains with OPCs using transplantation is a highly

relevant approach.
As a potential strategy to improve the chances of remyelination of chronic active lesions by transplanted OPCs, the authors focused on the interaction between Sema3A, a guidance molecule overexpressed in chronic MS lesions and a potent inhibitor of OPC recruitment and remyelination, and its receptor Nrp1. They hypothesized that deleting Sema3A receptor Nrp1 would render the hOPCs unresponsive to the chemorepellent effect of Sema3A and increase their recruitment to the lesions. They first generated a membrane bound GFP reporter-expressing hESC line, and then used CRISPR technology to delete Nrp1. Then they differentiated this line into OPCs. The authors demonstrate successful Nrp1 deletion that does not affect hOPC survival in vitro, but renders these cells resistant to the chemorepellent effect of Sema3A. They then undertake a series of very elegant in vivo experiments and demonstrate that transplanted cells survive for a long time in the host brains where they generate both myelinating oligodendrocytes and OPCs. They also demonstrate that these established OPCs are effectively mobilized towards demyelinating lesions even in old hosts, long after transplantation. They also show convincing evidence that Nrp1-/- cells reach areas of demyelination successfully even when Sema3A is overexpressed, which has a great translational relevance for MS. Similar results are obtained when hOPCs are transplanted contralaterally to the lesion 48h after induction of demyelination. In my opinion, this is a very important piece of work for the field of regenerative medicine as it re-introduces OPC transplantation as a strategy to promote remyelination, thus neuroprotection, in MS by suggesting means to modify transplanted cells to overcome the inhibitory cues present in MS lesions, previously considered as a major obstacle to the success of transplantation therapy.
While I am very impressed with the experiments performed, I have detected a few points that I believe should be addressed prior to publication:
1. relative to figure 2: immunocytochemical characterization of Nrp1+/+ and Nrp-/- cultures after exposure to Sema3A.
1A. It is not clear whether the graphs that indicate fold change with respect to untreated cells refer to absolute cell numbers or a percentage of positive cells within the total cells. This information is important for the readers to interpret the significance of the changes reported at 3 and 7 days of culture. At 3 days, there is a decrease in O4 expression after exposure of Nrp1+/+ cells to Sema 3A. The flow cytometry data indicate this is not due to cell death. No changes in proliferation were detected either. So, if O4+ cells decrease at 3 days of culture, do cells positive for other markers increase? Which ones? These do not appear to be PDGFralpha+ cells, so how is this interpreted? How does it fit with the data at 7 days that show no changes in MBP+ cells with exposure to Sema 3A?
1.B. How was the concentration of 5ug/mL chosen? (previously shown dose response??)
2. relative to Figure 3. Lesion +contralateral transplantation
2.A. It is indicated that : " Cell migration was assessed by the proportion of hOPCs moving towards or away from the lesion from the starting point of the injection site." It would be very helpful to provide information on how this injection site placement was defined in space (considering the spreading due to the injection, which area size surrounding the needle tract was considered as the starting point etc).

2.B. Early work in rats reported absence of OPC recruitment when cells were grafted at a distance from lesions in the normal rat spinal cord, but this could be overcome by X-irradiating the cord, presumably because this procedure damaged/depleted endogenous OPCs allowing for the transplanted cells to successfully migrate and establish within the tissue. Later work using transplanted human cells showed different results in that cells were migrating within normal tissue. What do the authors think might be the reason? Use of Rag mice (immunosuppression)? Specific properties of ES-derived cells that allow them to intrinsically "overcome" specific migration-inhibitory cues within the adult environment?

2.C. Related to this issue, on pages 9-10, the authors comment : " This is similar to previous work

demonstrating that transplanted rodent OPCs do not migrate in the adult rodent CNS without a stimulus such as demyelination" but the reference cited shows this migration to the lesion occurs ONLY in x-irradiated rat spinal cord, not in the normal spinal cord. Thus, this work should be cited properly and placed in the context potentially by highlighting different behavior of neonatal/rat/cell line versus embryonic/human cells after transplantation, or by highlighting different host environments (as different degrees of immunosuppression are always required when transplanting human cells, but not rodent cells or cell lines, which was the case in the reference cited)

3. With regard to the experiments evaluating the effect of the age of the host (LPC only without adding Sema3A), does Sema 3A expression increase in aged as compared to young mouse lesions?

4. On page 11: "The majority (~80%) of these human cells were OLIG2+ oligodendroglia, and over 50% were PDGFRα+ hOPCs (Figure 4C,D)"; so, what percentage were APC/CC1+? Is it expected that more than half of grafted cells will remain in the immature state rather than outcompeting the mouse cells during developmental myelination, which was suggested by MBP expression in shiverer brains in Supp Fig 1? (there could be a difference in outcompeting mouse OPCs in simple Rag mice as compared to Rag shiverer)

5. Page 12 and Supplementary Figure 4: The authors evaluate whether the recruitment capacity of transplanted hOPCs changes according to the host age. They show that the % of PDGFRalpha+Hu+ cells in the control lesion does not change in young vs old mice as a confirmation of intact capacity of transplanted embryonic hOPCs to reach the lesion. This is a very elegant proof. I would be curious on whether the hOPCs in 18m- old brains have also generated increased amount of mature oligodendrocytes and myelin (prior to lesion induction) with time (with respect to the young mice), in response to physiological stimuli, or whether their numbers progressively decrease.

6. On Page 12: "To test capacity for remyelination, we first transplanted NRP1+/+ or NRP1-/- cells directly into 48 hour old LPC-generated demyelinated lesions in Rag2-/- mice"- I find this a bit strange, but I assume that the wording may be an issue. To test remyelination, one would have to do the EM analyses, in this case combined with GFP immunogold labeling to identify human cells. What is strange about this logic is that the cells were directly transplanted into the lesion, rather than at the distance, given that the goal was to assess whether Nrp1 deletion can improve OPC recruitment and subsequent remyelination (in my opinion, this question was answered by the experiments using human OPC transplantation at P2 and lesions in adult). However, I find that the experiments with direct injection in the lesion are extremely useful to test whether intralesional Sema3A modifies human OPC differentiation (without confounding effects on OPC recruitment), as inhibitory effect was previously reported on rat OPCs (Syed et al., 2011), and if so, whether Nrp1 editing overcomes this inhibition. Thus, I suggest to slightly re-write this paragraph, focusing on testing the effect of Sema3A specifically on OPC differentiation in vivo rather than recruitment (as this is direct transplantation into the lesion) and then assess the proportion of human cells that express the mature oligo marker APC/CC1, or, if APC does not label well human cells, NogoA, previously used in human studies to label mature oligos.

7. Assessing CC1 expression would also be very useful in the later experiment with shiverer/rag mice at 3 weeks, which would indicate whether increased amount of MBP fluorescence observed corresponds to more new oligodendrocytes due to better recruitment (expected), or better remyelination by individual oligodendrocytes.

8. I think it would be also important to assess whether transplanted hOPCs give rise to cells other than oligodendroglia in the lesions (as well as in the normal tissue).

9. In the Figure 5F, it is difficult to judge myelin compaction. Higher magnification inset would be very useful/more convincing.

10. DISCUSSION

On page 15, in the 2nd paragraph that cites papers showing safety of human neural progenitor/OPC transplantation in MS, it would be appropriate to add the very recent reference on phase 1 clinical trial of allogeneic hNSC transplantation in human MS (intraventricular) (Leone et al., Cell Stem Cell).

The above-mentioned differences in transplanted cell migration through normal vs X-irradiated tissue (points 2b and 2C), and how these may or may not apply to human embryonic cells should be discussed.

Reviewer #3 (Remarks to the Author):

This paper reports the effect of knocking out neuropilin1 expression on human oligodendrocyte progenitors, as a means of ablating their responsiveness to semaphorin3A as a migratory stop signal. The authors propose that this strategy will promote OPC immigration into demyelinated lesions, from which they may otherwise be excluded because of high local sema3A expression. They support their hypothesis by engineering a neuropilin-1 knock-out line of human ESCs as their source of oligodendrocyte progenitor cells, and comparing the migration, differentiation and myelination of these cells to their Nrp1 wild-type counterparts in the lysolecithin-demyelinated lesions, using both neonatal (chimerization then demyelination) and adult (demyelination then transplantation) models.

The paper provides a first instance of genetically modifying human OPCs to improve their efficacy as therapeutic vectors. As such it provides an interesting and important addition to the literature and to the field. That said, some aspects of the data, their presentation and discussion thereof, could bear improvement. I’ve a number of suggestions thus intended to improve this work.

1. Nrp1 is a co-receptor with VEGFR2 of VEGF, and OPCs have been reported to express VEGFR2 and respond to VEGF, and VEGF-A has been reported to directly regulate OPC migration (PMID: 21775609) as well as proliferation (e.g., PMID: 3107564). Lysolecithin lesions may induce VEGF release, as this is a highly angiogenic, relatively hypoxic, environment. The authors should consider the effects of Nrp1 knock-out on the VEGF responsiveness of the OPCs – might this obscure or confound the effect of Nrp1 knock-out in ablating the inhibitory effect of Sema3A?
2. The fluorescent images are frequently weak (figures 2F, 2H, 4A, 4I; supplemental figs 5 and 6), with relatively low resolution and focus, and of too low a magnification to be really informative. Figures 4A and I in particular would benefit from higher resolution confocals.

3. The directionality data are not especially compelling; statistically significant, but hardly whopping effects. Granted there are many other pathways involved that may attenuate the relative contribution of the Nrp1-Sema3A interaction, and hence constrain the value of Nrp1 ablation. The authors’ arguments would be better served by focusing on their data showing the greater infiltration and myelination of the lysolecithin lesion by Nrp1 null OPCs.

4. What is the composition of the transplanted populations, and how enriched are they for OPCs and oligodendrocytes? None of the extant protocols for generating either OPCs or oligodendrocytes are perfect, and the Livesey protocol is not a widely used or characterized method, and focuses on oligodendrocytes rather than OPCs . Some flow cytometric or deeper immunocytochemical data

describing the composition of the transplanted cells – including off-target cells as well as just the OPC and oligo proportions - should be added.

In that regard, the authors do include a methods section (page 20) devoted to description of the single cell RNA-Seq analysis of their cells, but the only data provided are in supplemental table 1, and are limited to half-dozen differentially-expressed genes between the Nrp1 WT and null cells; also, it's not clear to me how these differential expression levels were determined. The authors state that "oligodendroglia were subsetted for downstream analysis," so I'd assume that these data were limited to the oligodendrocytes. But how were oligos defined as such? Some representations of the cluster here, its relative abundance, and violin plots of the marker genes are needed to qualify this population. Beyond that, what about the OPCs, neural precursors, astrocytes, etc? Any of these might also have been in the mix, and almost certainly were. What DEGs distinguished the Nrp1 null and WT cells for these phenotypes? Beyond these sparse RNA data, there's just a geo submission address to their raw data – which should not have to be reanalyzed from scratch by the casual reader. Overall, these data really need to be presented and discussed in much more detail; they may include sufficient data and integrated UMAPs to address the composition question, with note of both sample and cell number sizes, as well as more detail as to the age of the cells in vitro when captured for scRNA-Seq, and any corresponding flow data.

Absent such characterization, it is possible that persistent neural stem or progenitor cells might be the more responsive to Sema3A, and hence affected by Nrp1 ablation, with subsequent oligo differentiation after migration. While the net effect might be similar, the underlying biology would be very different.

5. While the limited RNA data (suppl table 1) showing little difference in RNA expression between Nrp1 WT and null oligodendroglia are reassuring, as are the similarities in proliferation rate and myelination, some greater qualification of genomic integrity and off-target edits post-CRISPR would be helpful. WGS with identification of potentially relevant variants has become more the standard for these types of data. At the very least, CGH array or similar assessment of chromosomal integrity and potential new structural variants is needed; scRNA-Seq alone can be blind to many processes that may affect translation and protein expression levels.

6. In that regard, more data should be provided as to how many passages the hESC line used had undergone, and how different that was in the CRISPR edited line. Most labs would incorporate CRISPR-only (no or irrelevant gRNA) controls with clonal re-expansion to control for the wide difference in passage number between WT and edited lines in this type of experiment. Absent such controls, WGS or an alternative prospective means of establishing post-editing genomic stability (e.g., GuideSeq, etc) should be used.

Minor points

1. It's often unclear whether the authors are referring to OPCs, oligodendrocytes, or both. This needs to be clarified throughout the text.

2. It's notable that using the Livesey protocol, the cells were caudalized by RA addition. Might this affect their migration biases in vivo in the callosal transplant model? Might OPCs not subjected to high initial RA have a different relative level of Nrp1 expression or different repertoire of Sema3A receptors, and respond differently to Sema3A? I recognize that this is beyond the scope of the authors present work, but it does warrant brief coverage in their Discussion.

3. Some verification of the persistence and duration thereof of bioactive Sema3A after tissue injection

is needed, especially in the injections into lyso lesions at 48 hrs (as in figure 3). The post-lysolecithin lesion environment is highly proteolytic; how confident are the authors as to the levels and activity of Sema3A that persist?

4. In that regard, lysolecithin best models acute demyelination; it is a suboptimal model for the chronic effects of a demyelinating injury, with the astrogliosis, microglial infiltration, and variable axonal loss that attend these lesions. It doesn’t necessarily reflect very well the environment into which modified OPCs would be delivered in patients. The authors should discuss these limitations of their model, and also discuss alternative models for focal demyelination (ethidium/RT, short cuprizone, Theiler’s, etc.) that might have been used, and their specific reasons for using lysolecithin.

5. The authors in several instances state that their work will “reignite” interest and activity in the use of OPC transplantation as a therapeutic strategy. It’s not clear to me that such interest was ever extinguished, to be so reignited – this has been, and remains, an active field of investigation across many labs. The authors claim to be reigniting the field seems a bit overstated, and would really best be toned down.

Dear Editor and reviewers,

We thank the reviewers for their comments on our article NCOMMS-23-56625, which we believe we have addressed in full below and in our revised article version. We think these comments have improved the paper and hope it will now be deemed suitable for publishing in Nature Communications. We address each point below and changes are highlighted on the accompanying manuscript.

Reviewer #1 (Remarks to the Author):

In this manuscript, Wagstaff et al. generated human OPCs from human embryonic stem cells and introduced them to young and aged mice that undergo lysolecithin-induced focal demyelination. The experiments demonstrate that hOPCs injected into the mouse corpus callosum are capable of surviving and migrating along the corpus callosum into areas with demyelination. My main concern with the manuscript, given its primary focus on the therapeutic potential of transplanting hOPCs for remyelination, lies in its relevance to human demyelinating disease. As it stands, the data convincingly demonstrate that deleting NRP1 from OPCs blocks their responsiveness to Sema3A, but not that Sema3A contributes to inefficient remyelination in demyelinating disease. Major concerns are described below:

Major:

- Overall, the data presented here indicate that the artificial addition of Sema3A is required for the knockout of NRP1 to influence OPC migration into LPC lesions in mice. While it may be the case that chronic lesions in humans contain higher levels of Sema3A than rodent LPC injections, it is not clear from the data presented in this paper whether Sema3A is a critical inhibitor of remyelination in that context.

There is good evidence that SEMA3A is one of critical factors preventing remyelination in MS as we and others have shown previously. We summarise this evidence here and have added more background on this in our article to make it clearer (results section on page 6).

There is more *SEMA3A* RNA expression in human MS white matter lesions as seen by both in situ hybridisation (Williams et al., 2007) and single nuclei RNAseq (Macnair et al., 2023) than control non-MS tissue. Furthermore, Chronic active (CA) lesions, in particular, express more SEMA3A protein, and have fewer OPCs detectable within them, as shown in our previous article (Boyd et al., 2013), and presence of these lesions correlate with increased disability and reduced remyelination in people with MS (Absinta et al., 2019, Wittayer et al., 2022). This, together with preclinical data identifying Sema3A as an inhibitor of OPC migration in development and injury (Spassky et al., 2002, Williams et al., 2007, Syed et al., 2011, Piaton et al., 2011, Boyd et al., 2013) made SEMA3A a good choice to carry out this proof-of-concept experiment that editing OPCs to remove the SEMA3A receptor would improve their remyelination potential.

It would be naïve to think that this molecule is the only factor or even the most important factor preventing remyelination. However, our point here is also to show as a proof of principle that modulating the response to one such factor in transplanted OPCs can aid remyelination. We suggest in our discussion that altering several such pathways in combination may be even more effective.

References mentioned in this response (already in article):

Absinta, M. *et al.* Association of Chronic Active Multiple Sclerosis Lesions With Disability In Vivo. *JAMA Neurology* **76**, 1474-1483, doi:10.1001/jamaneurol.2019.2399 (2019).

Boyd, A., Zhang, H. & Williams, A. Insufficient OPC migration into demyelinated lesions is a cause of poor remyelination in MS and mouse models. *Acta Neuropathol* **125**, 841-859, doi:10.1007/s00401-013-1112-y (2013).

Macnair, W. *et al.* Single nuclei RNAseq stratifies multiple sclerosis patients into distinct white matter glial responses. *bioRxiv*, 2022.2004.2006.487263, doi:10.1101/2022.04.06.487263 (2023).

Piaton, G. *et al.* Class 3 semaphorins influence oligodendrocyte precursor recruitment and remyelination in adult central nervous system. *Brain* **134**, 1156-1167, doi:10.1093/brain/awr022 (2011).

Spassky, N. *et al.* Directional Guidance of Oligodendroglial Migration by Class 3 Semaphorins and Netrin-1. *The Journal of Neuroscience* **22**, 5992-6004, doi:10.1523/jneurosci.22-14-05992.2002 (2002).

Syed, Y. A. *et al.* Inhibition of CNS remyelination by the presence of semaphorin 3A. *J Neurosci* **31**, 3719-3728, doi:10.1523/jneurosci.4930-10.2011 (2011).

Williams, A. *et al.* Semaphorin 3A and 3F: key players in myelin repair in multiple sclerosis? *Brain* **130**, 2554-2565, doi:10.1093/brain/awm202 (2007).

Wittayer, M. *et al.* Spatial distribution of multiple sclerosis iron rim lesions and their impact on disability. *Multiple Sclerosis and Related Disorders* **64**, 103967, doi:https://doi.org/10.1016/j.msard.2022.103967 (2022).

- The authors also mention that ageing contributes to reduced recruitment of OPCs to lesions, but their data in Figure 3E, F, and S4B show no difference in recruitment between young and old mice, which suggests that LPC-induced demyelination in mice may not be a suitable model for studying ageing-related factors that reduce OPC recruitment. Perhaps a model more similar to human demyelination, such as EAE, could better recapitulate some of the factors limiting OPC recruitment to lesions in human demyelinating disease that the authors hope to alleviate with the knockout of NRP1.

There is indeed evidence that ageing contributes to reduced recruitment of OPCs to lesions, but this evidence is from aged endogenous rodent OPCs in an aged rodent environment. Our data show that transplanted ES-derived human OPCs (by definition, "young") were recruited similarly to lesions in young and old mouse environments. This suggests that human OPC recruitment is dependent on their intrinsic properties, in this context, more than the environment they pass though – which we find of interest both to understanding the biology and of promise for future potential translation. We have added a sentence to emphasise this (results section page 11).

We do not believe that use of a different model would help further. EAE is a T-cell driven acute spinal cord inflammatory and demyelinating model, used best and very successfully in the field to study prevention/treatment of the adaptive immune-driven aspect of MS, rather than the remyelinating/neuroprotective phase of MS.

- **The rationale for injecting hOPCs perinatally is unclear from a therapeutic perspective.**

The rationale here was simply to look at longer term engraftment of transplanted cells to see if they could respond effectively even months after their transplantation. From a therapeutic perspective, we clearly would not advocate perinatal OPC transplantation in humans. However, this experiment suggests that cells transplanted weeks before demyelinating lesions are generated can still respond favourably to the lesion. Therapeutically, this may mean that transplanted OPCs may be useful over a longer period of time - potentially useful in a chronic disease where new lesions occur over time, avoiding the need for multiple OPC transplants. We have altered the wording to make this clear on page 11.

- **What are the hESC-derived cells that don't express PDGFRa or Olig2? Some attempt to characterize the identity of all the injected cells would be useful for interpretation.**

We have now added transcriptomic data to better characterise all of these cells (see Supplemental Figure 2) in addition to the immunofluorescence characterisation of the oligodendroglia only, shown in Figure 1D,E.

By scRNAseq, we find that the injected cells are mostly a mixture of astrocytes (~50%) and oligodendroglia (~30%), with the remaining being other cells less differentiated and without a clear gene signature (termed other CNS cells) (Supplemental Figure 2 A-C). These have been identified using classical marker genes (Supplemental Figure 2 B). The proportions of these are not different between the NRP1-/- and NRP1+/+ cells (Supplemental Figure 2 C). (We reproduce Supplemental figure 2A-C below)

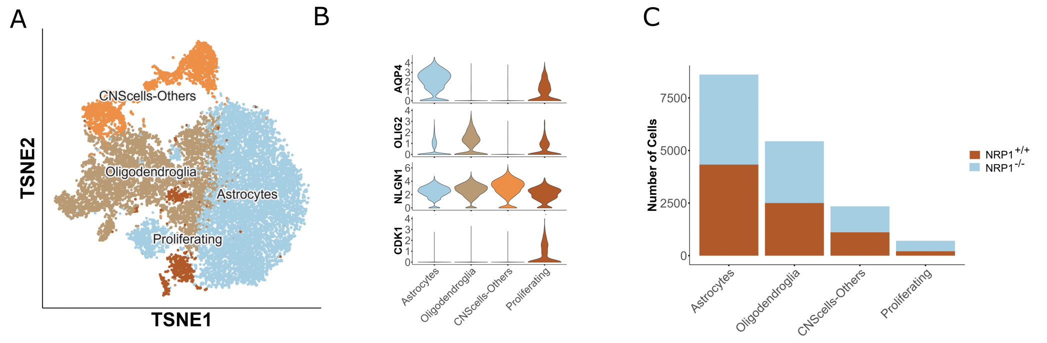

- **What is the % of mature oligodendrocytes that arose from injected cells? Is this % more or less in the NRP1-/- cells, and does that lead to an overall higher number of mature OLs in the corpus callosum?**

To determine the percentage of mature oligodendrocytes arising from these injected cells, we performed immunofluorescence on tissue from Rag2$^{-/-}$ mice 6 weeks after transplants of either NRP1$^{-/-}$ or NRP1$^{+/+}$ cells (but without demyelination). We found that most human (hNu+) cells remaining at this timepoint were OLIG2+ oligodendroglia, with a larger proportion of these remaining as PDGFRA+ OPCs than CC1+ mature oligodendrocytes, and the remainder being GFAP+ astrocytes. There was no significant difference in proportions between NRP1$^{-/-}$ or NRP1$^{+/+}$ cells and the cells are evenly distributed between right and left hemispheres. We have added this to Figure 4 and Supplemental Figure 4 (relevant part reproduced below).

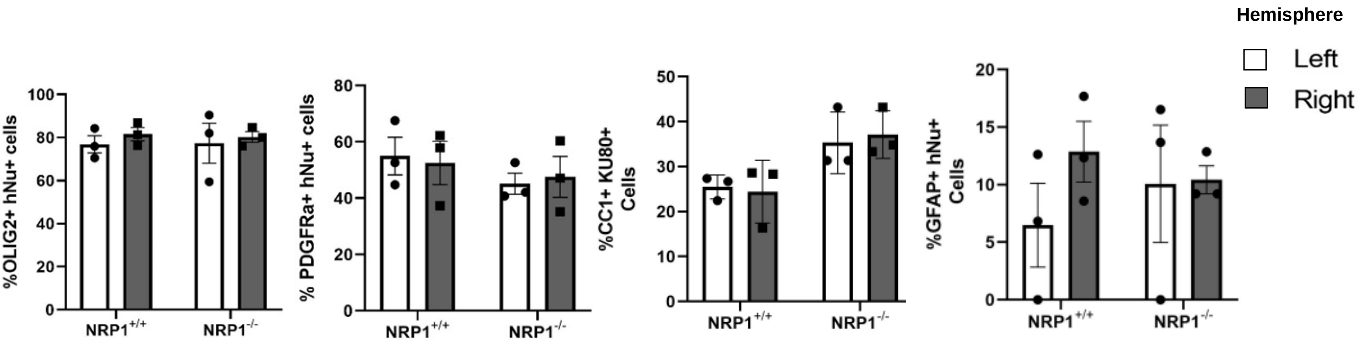

Minor:

- **In Figure S1E, why is there little overlap between GFP and MBP? Seems like the majority of GFP+ sheaths are not MBP+.**

We now include a supplementary video (new Supplementary Video 1), of a Z-stack showing that MBP+ myelin sheaths are surrounded by GFP immunostaining, suggesting that compact myelin is excluding the GFP but the abaxonal myelin membrane (outer myelin sheath wrap) expresses GFP. We hypothesise that this is as GFP is a large molecule. We also know that all MBP+ immunofluorescence staining in the *Shiverer* mouse is derived from human cells.

- **More zoomed out views of the corpus callosum (similar to Figure 3B) should be included in Figure S2.**

We have added these as requested, now as Supplemental Figure 3.

- Figure 3I should include example images from a Sema3A lesion injected with NRP1-/- cells.

We think the reviewer means Figure 4I and have added this as requested (now Figure 4 J).

Reviewer #2 (Remarks to the Author):

The manuscript by Wagstaff and colleagues has investigated the utility of genetic modification of human OPCs (hOPCs) prior to transplantation in overcoming the non-permissive environment of chronic active lesions to achieve remyelination in multiple sclerosis. This is an extremely important issue given that clinical trials of drugs that promote differentiation of endogenous OPCs have not shown major benefits in patients with MS, likely due to paucity of OPCs in chronic lesions, age-induced lack of responsiveness to pro-differentiating stimuli, as well as limited access of these drugs to the lesions. These issues, in addition to the fact that low oligodendroglial numbers are associated with MS progression, strongly indicate that repopulating MS brains with OPCs using transplantation is a highly relevant approach. As a potential strategy to improve the chances of remyelination of chronic active lesions by transplanted OPCs, the authors focused on the interaction between Sema3A, a guidance molecule overexpressed in chronic MS lesions and a potent inhibitor of OPC recruitment and remyelination, and its receptor Nrp1. They hypothesized that deleting Sema3A receptor Nrp1 would render the hOPCs unresponsive to the chemorepellent effect of Sema3A and increase their recruitment to the lesions. They first generated a membrane bound GFP reporter-expressing hESC line, and then used CRISPR technology to delete Nrp1. Then they differentiated this line into OPCs. The authors demonstrate successful Nrp1 deletion that does not affect hOPC survival in vitro, but renders these cells resistant to the chemorepellent effect of Sema3A. They then undertake a series of very elegant in vivo experiments and demonstrate that transplanted cells survive for a long time in the host brains where they generate both myelinating oligodendrocytes and OPCs. They also demonstrate that these established OPCs are effectively mobilized towards demyelinating lesions even in old hosts, long after transplantation. They also show convincing evidence that Nrp1-/- cells reach areas of demyelination successfully even when Sema3A is overexpressed, which has a great translational relevance for MS. Similar results are obtained when hOPCs are transplanted contralaterally to the lesion 48h after induction of demyelination. In my opinion, this is a very important piece of work for the field of regenerative medicine as it re-introduces OPC transplantation as a strategy to promote remyelination, thus neuroprotection, in MS by suggesting means to modify transplanted cells to overcome the inhibitory cues present in MS lesions, previously considered as a major obstacle to the success of transplantation therapy. While I am very impressed with the experiments performed, I have detected a few points that I believe should be addressed prior to publication:

1. relative to figure 2: immunocytochemical characterization of Nrp1+/+ and Nrp-/- cultures after exposure to Sema3A.

1A. It is not clear whether the graphs that indicate fold change with respect to untreated cells refer to absolute cell numbers or a percentage of positive cells within the total cells. This information is important for the readers to interpret the significance of the changes reported at 3 and 7 days of culture.

We have used percentage of OLIG2+ cells in the in vitro culture work to calculate the fold change as there is variation in the number of OLIG2+ cells in each differentiation culture and we have added this to the methods section (page 28)

At 3 days, there is a decrease in O4 expression after exposure of Nrp1+/+ cells to Sema 3A. The flow cytometry data indicate this is not due to cell death. No changes in proliferation were detected either. So, if O4+ cells decrease at 3 days of culture, do cells positive for other markers increase? Which ones? These do not appear to be PDGFralpha+ cells, so how is this interpreted? How does it fit with the data at 7 days that show no changes in MBP+ cells with exposure to Sema 3A?

The finding of reduced O4+ cells after SEMA3A treatment in vitro is robust, having been first reported by the Kotter lab (Syed et al., 2011) but also seen previously in experiments in our lab. The explanation in the published article is that SEMA3A treatment causes an increase in the A2B5+ cells with fewer processes and a decrease in MBP transcript expression at this early time-point (they report at 2 days). However, when the cells are cultured until the 7-day time-point, in differentiation medium, the MBP protein expression catches up, presumably as differentiation medium contains many drivers of OPC differentiation.

Syed, Y. A. *et al.* Inhibition of CNS remyelination by the presence of semaphorin 3A. *J Neurosci* **31**, 3719-3728, doi:10.1523/jneurosci.4930-10.2011 (2011).

1.B. How was the concentration of 5ug/mL chosen? (previously shown dose response??)

This concentration was taken as the middle dose which was effective for OPCs from a dose response experiment performed in the Syed et al., 2011 paper, which in turn was over a range used to stimulate in vitro neuronal dendrite growth (Schlomann et al., 2009). We have added this information to the manuscript. (Page 8)

Syed, Y. A. *et al.* Inhibition of CNS remyelination by the presence of semaphorin 3A. *J Neurosci* **31**, 3719-3728, doi:10.1523/jneurosci.4930-10.2011 (2011).

Schlomann U, et al.. The stimulation of dendrite growth by Sema3A requires integrin engagement and focal adhesion kinase. J Cell Sci. 2009 Jun 15;122(Pt 12):2034-42. doi: 10.1242/jcs.038232.

2. relative to Figure 3. Lesion +contralateral transplantation
2.A. It is indicated that : " Cell migration was assessed by the proportion of hOPCs moving towards or away from the lesion from the starting point of the injection site." It would be very helpful to provide information on how this injection site placement was defined in space (considering the spreading due to the injection, which area size surrounding the needle tract was considered as the starting point etc).

The coordinates of the injection were as follows: (1.2mm antero-posterior and 1mm lateral from the bregma at a depth of 1.4mm, on right). We then identified the starting point on the histological section by locating the bottom of the needle tract which is visible by histology/bright field. We have now added this to the manuscript (page 9).

2.B. Early work in rats reported absence of OPC recruitment when cells were grafted at a distance from lesions in the normal rat spinal cord, but this could be overcome by X-irradiating the cord, presumably because this procedure damaged/depleted endogenous OPCs allowing for the transplanted cells to successfully migrate and establish within the tissue. Later work using transplanted human cells showed different results in that cells were migrating within normal tissue. What do the authors think might be the reason? Use of Rag mice (immunosuppression)? Specific properties of ES-derived cells that allow them to intrinsically "overcome" specific migration-inhibitory cues within the adult environment?

Our data supports the previous evidence that OPCs transplanted into adult rodents do not migrate in normal tissue without a stimulus (in our case a demyelinated lesion) Without a demyelinated lesion, cells simply accumulate around the injection site (see Supplemental Figure 3).

However, OPCs transplanted into mouse pups are able to migrate through the developing mouse brain – as shown in our experiments (e.g. Figure 4) and in seminal papers by the Steve Goldman group (e.g. Windrem et al., 2008). It may be that successful OPC migration in development but not adulthood relates to presence of directional cues important in development to complete myelination, but the absence of these cues in normal adulthood when myelination is complete.

Windrem, M. S. *et al*. Neonatal chimerization with human glial progenitor cells can both remyelinate and rescue the otherwise lethally hypomyelinated shiverer mouse. *Cell Stem Cell* **2**, 553-565, doi:10.1016/j.stem.2008.03.020 (2008).

2.C. Related to this issue, on pages 9-10, the authors comment : " This is similar to previous work demonstrating that transplanted rodent OPCs do not migrate in the adult rodent CNS without a stimulus such as demyelination" but the reference cited shows this migration to the lesion occurs ONLY in x-irradiated rat spinal cord, not in the normal spinal cord. Thus, this work should be cited properly and placed in the context potentially by highlighting different behavior of neonatal/rat/cell line versus embryonic/human cells after transplantation, or by highlighting different host environments (as different degrees of immunosuppression are always required when transplanting human cells, but not rodent cells or cell lines, which was the case in the reference cited)

Thank you - we have altered the wording of this part to correct this (page 10) and addressed this further in the discussion section as we agree it is important (page 16-17).

3. With regard to the experiments evaluating the effect of the age of the host (LPC only without adding Sema3A), does Sema 3A expression increase in aged as compared to young mouse lesions?

We had the same thought and tried testing this using a western blot from tissue from the lesional areas in young and aged mice. The available antibody is not in any way ideal, and we would not be keen to add this to the paper due to this, but we include it here but do not think this showed a detectable difference. In keeping with this, and with evidence that we have more confidence in, we did not see a change in the recruitment of cells to the lesion in aged compared to young mice.

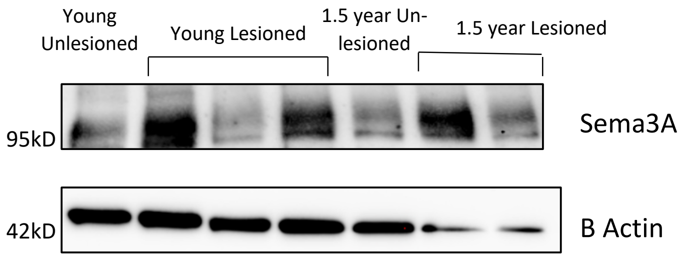

4. On page 11: "The majority (~80%) of these human cells were OLIG2+ oligodendroglia, and over 50% were PDGFRα+ hOPCs (Figure 4C,D)"; so, what percentage were APC/CC1+? Is it expected that more than half of grafted cells will remain in the immature state rather than outcompeting the mouse cells during developmental myelination, which was suggested by MBP expression in shiverer brains in Supp Fig 1? (there could be a difference in outcompeting mouse OPCs in simple Rag mice as compared to Rag shiverer)

We have now added a graph of the CC1+ oligodendroglia to Figure 4 (new Figure 4 E) showing that in the Rag2-/- mice, around 30% of the transplanted human cells (HuN+) were CC1+ oligodendrocytes and the remainder of the human cells are GFAP+ astrocytes (Supplemental Figure 4).

We also looked in the *Shi/Shi:Rag2-/-* mice, and found that around 30% of the transplanted human cells (HuN+) also became CC1+ oligodendrocytes at the 6 week time point after transplantation (see below and new Figure 5 C). The MBP expression in the *Shiverer* mice looks very obvious simply as there is no mouse MBP staining due to the *Shiverer* mutation.

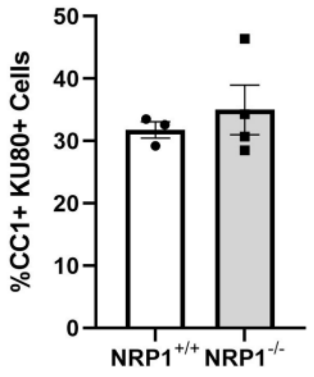

% of transplanted human cells that become CC1+ in *Shi/Shi:Rag2-/-*

Comparison of the transplants into *Rag2-/-* and the *Shi/Shi:Rag2-/-* mice showed no significant difference in the proportion of transplanted cells that became CC1+ oligodendrocytes as shown below. (n=3 for each condition, two-tailed unpaired t-test, p=0.0545)

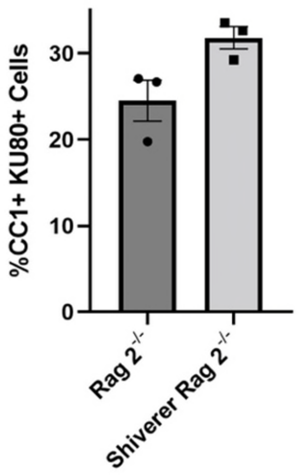

5. Page 12 and Supplementary Figure 4: The authors evaluate whether the recruitment capacity of transplanted hOPCs changes according to the host age. They show that the % of PDGFRalpha+Hu+ cells in the control lesion does not change in young vs old mice as a confirmation of intact capacity of transplanted embryonic hOPCs to reach the lesion. This is a very elegant proof. I would be curious on whether the hOPCs in 18m- old brains have also generated increased amount of mature oligodendrocytes and myelin (prior to lesion induction) with time (with respect to the young mice), in response to physiological stimuli, or whether their numbers progressively decrease.

Thank you for this. We would also be interested in whether hOPCs in 18 month brains in unlesioned mice behave differently in response to aged environment. However, unfortunately, we do not have tissue available for this already and could not generate it in the timescale for revisions.

6. On Page 12: "To test capacity for remyelination, we first transplanted NRP1+/+ or

NRP1-/- cells directly into 48 hour old LPC-generated demyelinated lesions in Rag2-/- mice"- I find this a bit strange, but I assume that the wording may be an issue. To test remyelination, one would have to do the EM analyses, in this case combined with GFP immunogold labeling to identify human cells. What is strange about this logic is that the cells were directly transplanted into the lesion, rather than at the distance, given that the goal was to assess whether Nrp1 deletion can improve OPC recruitment and subsequent remyelination (in my opinion, this question was answered by the experiments using human OPC transplantation at P2 and lesions in adult). However, I find that the experiments with direct injection in the lesion are extremely useful to test whether intralesional Sema3A modifies human OPC differentiation (without confounding effects on OPC recruitment), as inhibitory effect was previously reported on rat OPCs (Syed et al., 2011), and if so, whether Nrp1 editing overcomes this inhibition. Thus, I suggest to slightly re-write this paragraph, focusing on testing the effect of Sema3A specifically on OPC differentiation in vivo rather than recruitment (as this is direct transplantation into the lesion) and then assess the proportion of human cells that express the mature oligo marker APC/CC1, or, if APC does not label well human cells, NogoA, previously used in human studies to label mature oligos.

Thank you for this help as we accept this was somewhat confusing. We agree that this experiment does not add anything to the article and as SEMA3A was not added to the lesion in this experiment, we have decided to remove this figure.

7. Assessing CC1 expression would also be very useful in the later experiment with shiverer/rag mice at 3 weeks, which would indicate whether increased amount of MBP fluorescence observed corresponds to more new oligodendrocytes due to better recruitment (expected), or better remyelination by individual oligodendrocytes.

We agree this is a good addition and have now assessed the proportion of human transplanted cells which were CC1+at 3 weeks after transplantation of NRP1$^{-/-}$ or NRP1$^{+/+}$ cells (Supplemental Figure 7 E). At this timepoint, the number of CC1+ cells was equal between NRP1$^{-/-}$ and NRP1$^{+/+}$ transplants, suggesting a degree of catch up in cell number but not myelin produced at this later timepoint. This may relate to a later decline in lesional SEMA3A expression.

8. I think it would be also important to assess whether transplanted hOPCs give rise to cells other than oligodendroglia in the lesions (as well as in the normal tissue). In response to this and Reviewer 1's similar comment, we have now added this. The data are presented in *Rag2*$^{-/-}$ brains without lesions (Figure 4 B-E, Supplemental Figure 4), in *Shi/Shi:Rag2-/-* brains without lesions (Figure 5 C, Supplemental Figure 7 B) and in *Shi/Shi:Rag2-/-* brains with lesions (Supplemental Figure 7 E,F).

9. In the Figure 5F, it is difficult to judge myelin compaction. Higher magnification inset would be very useful/more convincing.

We have added a higher magnification inset as requested becoming new Figure 5G.

10. DISCUSSION

On page 15, in the 2nd paragraph that cites papers showing safety of human neural progenitor/OPC transplantation in MS, it would be appropriate to add the very recent reference on phase 1 clinical trial of allogeneic hNSC transplantation in human MS (intraventricular) (Leone et al., Cell Stem Cell).

We agree and were excited by this paper, which was published after we submitted our paper (added to discussion on page 16).

The above-mentioned differences in transplanted cell migration through normal vs X-irradiated tissue (points 2b and 2C), and how these may or may not apply to human embryonic cells should be discussed.

Thank you – we have added this to the discussion on page 16-17.

Reviewer #3 (Remarks to the Author):

This paper reports the effect of knocking out neuropilin1 expression on human oligodendrocyte progenitors, as a means of ablating their responsiveness to semaphorin3A as a migratory stop signal. The authors propose that this strategy will promote OPC immigration into demyelinated lesions, from which they may otherwise be excluded because of high local sema3A expression. They support their hypothesis by engineering a neuropilin-1 knock-out line of human ESCs as their source of oligodendrocyte progenitor cells, and comparing the migration, differentiation and myelination of these cells to their Nrp1 wild-type counterparts in the lysolecithin-demyelinated lesions, using both neonatal (chimerization then demyelination) and adult (demyelination then transplantation) models.

The paper provides a first instance of genetically modifying human OPCs to improve their efficacy as therapeutic vectors. As such it provides an interesting and important addition to the literature and to the field. That said, some aspects of the data, their presentation and discussion thereof, could bear improvement. I've a number of suggestions thus intended to improve this work.

1. Nrp1 is a co-receptor with VEGFR2 of VEGF, and OPCs have been reported to express VEGFR2 and respond to VEGF, and VEGF-A has been reported to directly regulate OPC

migration (PMID: 21775609) as well as proliferation (e.g., PMID: 3107564). Lysolecithin lesions may induce VEGF release, as this is a highly angiogenic, relatively hypoxic, environment. The authors should consider the effects of Nrp1 knock-out on the VEGF responsiveness of the OPCs – might this obscure or confound the effect of Nrp1 knock-out in ablating the inhibitory effect of Sema3A?

VEGF does indeed bind NRP1 directly as well as VEGFR2 which is expressed on OPCs and NRP1 dimerises with VEGFR2. Knocking out the NRP1 receptor will prevent both SEMA3A and VEGF signalling through NRP1 and reduce the efficiency of the action of VEGF through VEGFR2 binding. VEGF is shown to be a chemoattractant in vitro and work from the Durbec lab (Cayre et al., 2013) shows that LPC-induced lesions are angiogenic and that use of anti-VEGF blocking antibody reduced both angiogenesis and precursor recruitment. Thus, loss of VEGF signalling would be expected to decrease OPC recruitment, whereas loss of SEMA3A signalling through NRP1 would be expected to increase OPC recruitment. Transgenic mice designed to lack the binding site for Sema3A on the NRP1 receptor, but retaining the VEGF binding site and function, show enhanced OPC recruitment to a LPC demyelinated lesion (Piaton et al., 2011). probably through both loss of Sema3A inhibition and retention of VEGF chemoattraction.

In our experiment, we see increased OPCs in the lesions, which may indeed be more marked still if we had been able to edit out only the SEMA3-binding site of NRP1. We have added a comment mentioning this in the discussion page 15.

Cayre M, *et al.* Netrin 1 contributes to vascular remodeling in the subventricular zone and promotes progenitor emigration after demyelination. Development. 2013 Aug;140(15):3107-17. doi: 10.1242/dev.092999. Epub 2013 Jul 3. PMID: 23824572.

Piaton, G. *et al.* Class 3 semaphorins influence oligodendrocyte precursor recruitment and remyelination in adult central nervous system. *Brain* **134**, 1156-1167, doi:10.1093/brain/awr022 (2011).

2. The fluorescent images are frequently weak (figures 2F, 2H, 4A, 4I; supplemental figs 5 and 6), with relatively low resolution and focus, and of too low a magnification to be really informative. Figures 4A and I in particular would benefit from higher resolution confocals.

We wonder if some of the problem here is related to the conversion of images into the PDF – the high resolution files are also available for download . However, we have taken this opportunity to improve the figures further and have placed insets into Figure 5 and Supplemental Figure 3 to try and help.

3. The directionality data are not especially compelling; statistically significant, but hardly whopping effects. Granted there are many other pathways involved that may attenuate the relative contribution of the Nrp1-Sema3A interaction, and hence constrain the value of Nrp1 ablation. The authors' arguments would be better served by focusing

on their data showing the greater infiltration and myelination of the lysolecithin lesion by Nrp1 null OPCs.

We agree that, generally, in vitro transwell experiments are often underwhelming. We also agree that the most important results are the in vivo recruitment and myelination results.

4. What is the composition of the transplanted populations, and how enriched are they for OPCs and oligodendrocytes? None of the extant protocols for generating either OPCs or oligodendrocytes are perfect, and the Livesey protocol is not a widely used or characterized method, and focuses on oligodendrocytes rather than OPCs . Some flow cytometric or deeper immunocytochemical data describing the composition of the transplanted cells – including off-target cells as well as just the OPC and oligo proportions - should be added.

In that regard, the authors do include a methods section (page 20) devoted to description of the single cell RNA-Seq analysis of their cells, but the only data provided are in supplemental table 1, and are limited to half-dozen differentially-expressed genes between the Nrp1 WT and null cells; also, it's not clear to me how these differential expression levels were determined. The authors state that "oligodendroglia were subsetted for downstream analysis," so I'd assume that these data were limited to the oligodendrocytes. But how were oligos defined as such? Some representations of the cluster here, its relative abundance, and violin plots of the marker genes are needed to qualify this population. Beyond that, what about the OPCs, neural precursors, astrocytes, etc? Any of these might also have been in the mix, and almost certainly were. What DEGs distinguished the Nrp1 null and WT cells for these phenotypes? Beyond these sparse RNA data, there's just a geo submission address to their raw data – which should not have to be reanalyzed from scratch by the casual reader. Overall, these data really need to be presented and discussed in much more detail; they may include sufficient data and integrated UMAPs to address the composition question, with note of both sample and cell number sizes, as well as more detail as to the age of the cells in vitro when captured for scRNA-Seq, and any corresponding flow data.

Absent such characterization, it is possible that persistent neural stem or progenitor cells might be the more responsive to Sema3A, and hence affected by Nrp1 ablation, with subsequent oligo differentiation after migration. While the net effect might be similar, the underlying biology would be very different.

To address this, and Reviewer 1's similar comment, we have now added a supplemental figure describing the transplant cell populations at the transcriptomic (scRNAseq) level more fully (Supplemental Figure 2). We include violin plots with marker genes of our cell type clusters (Supplemental Figure 2 B) The composition is shown in (Supplemental figure 2 C).

We noted it was unclear that we had selected both oligodendrocytes and oligodendrocyte precursor cells (OPCs) for the differential expression, so we decided to be more precise and select the OPCs only, as it is the most relevant to the study. We add details on how the OPCs were characterised and subsetted, showing a feature plot with the subsetted OPC population, which we defined as the cluster of cells with the highest expression of *PDGFRA* (Supplemental Figure 2 D-E). We then performed differential gene expression between NRP1$^{-/-}$ and NRP1$^{+/+}$ OPCs, now shown as a volcano plot (Supplemental Figure 2 F). Differential gene expression for OPCs and the other cell-types are included in Supplementary tables 2-5. The differential expression levels were kept with the default parameters from the Seurat's FindMarkers function (0.25 logFC and 0.1 pct cells expressing the gene) and this has now been added to the methods. Thank you for noticing that we had not added the timing of the transcriptomic experiment or the number of cells – this is now corrected in the methods (Page 21-22)

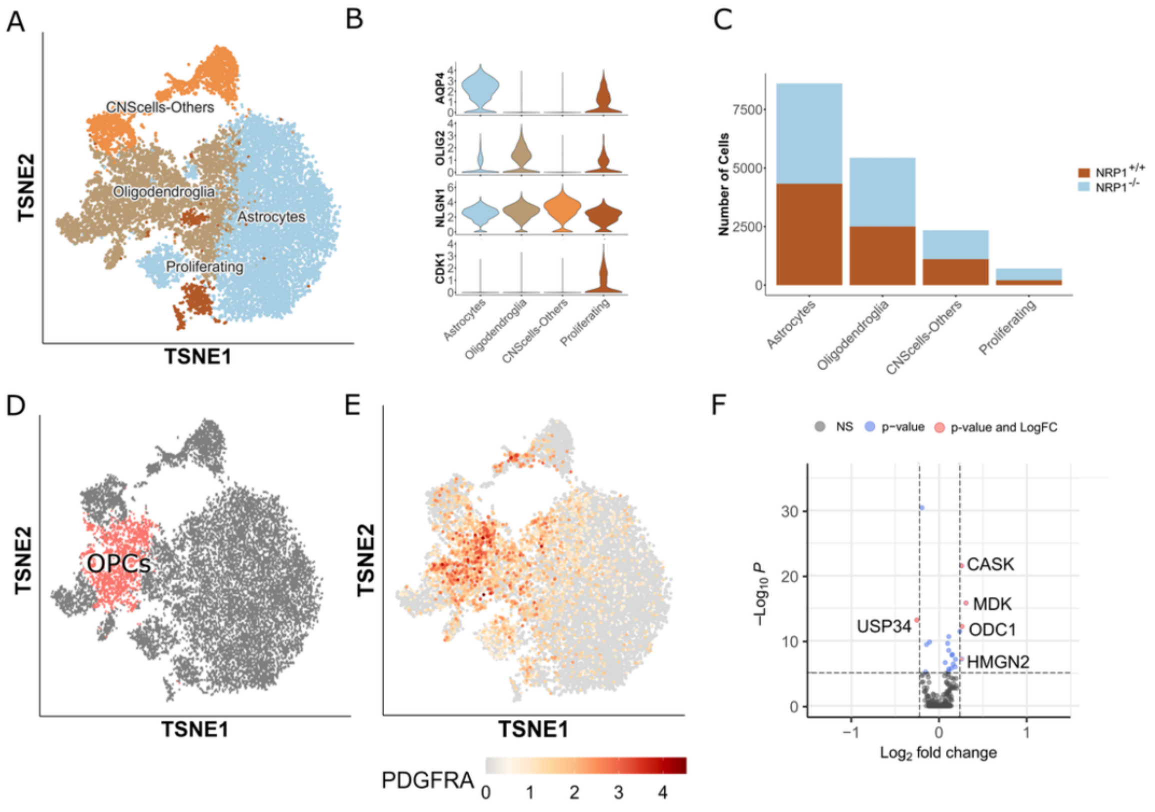

5. While the limited RNA data (suppl table 1) showing little difference in RNA expression between Nrp1 WT and null oligodendroglia are reassuring, as are the similarities in proliferation rate and myelination, some greater qualification of genomic integrity and off-target edits post-CRISPR would be helpful. WGS with identification of potentially relevant variants has become more the standard for these types of data. At the very least, CGH array or similar assessment of chromosomal integrity and potential new structural variants is needed; scRNA-Seq alone can be blind to many processes that may affect translation and protein expression levels.

We were also reassured by the limited differences in RNA expression and functional similarities in vitro. However, we accept that other differences can occur which are not obvious from this or scRNAseq. We took the reviewer's advice and performed SNP analysis of the cells on the hES cells. These data are presented in the Supplemental table 1, which shows that the karyotype is unchanged. The CNV analysis shows there are some CNVs present in NRP1$^{-/-}$ cells, but we focussed on the small number that were not also present in the NRP1$^{+/+}$. None of these were in areas predicted as potential gRNA off-targets. We next investigated the genes within the duplicated/lost areas to see whether these led to transcript expression differences in our scRNAseq dataset generally. The only visible changes were the decreased expression of USP34 and XPO1 (both on chromosome 2) in the NRP1$^{-/-}$ cells. From our scRNAseq analysis of OPCs, only expression of USP34 (a deubiquitinase) is significantly reduced in NRP1$^{-/-}$ OPCs, which have lost a copy of this gene. (Supplemental Figure 2 G and reproduced below).

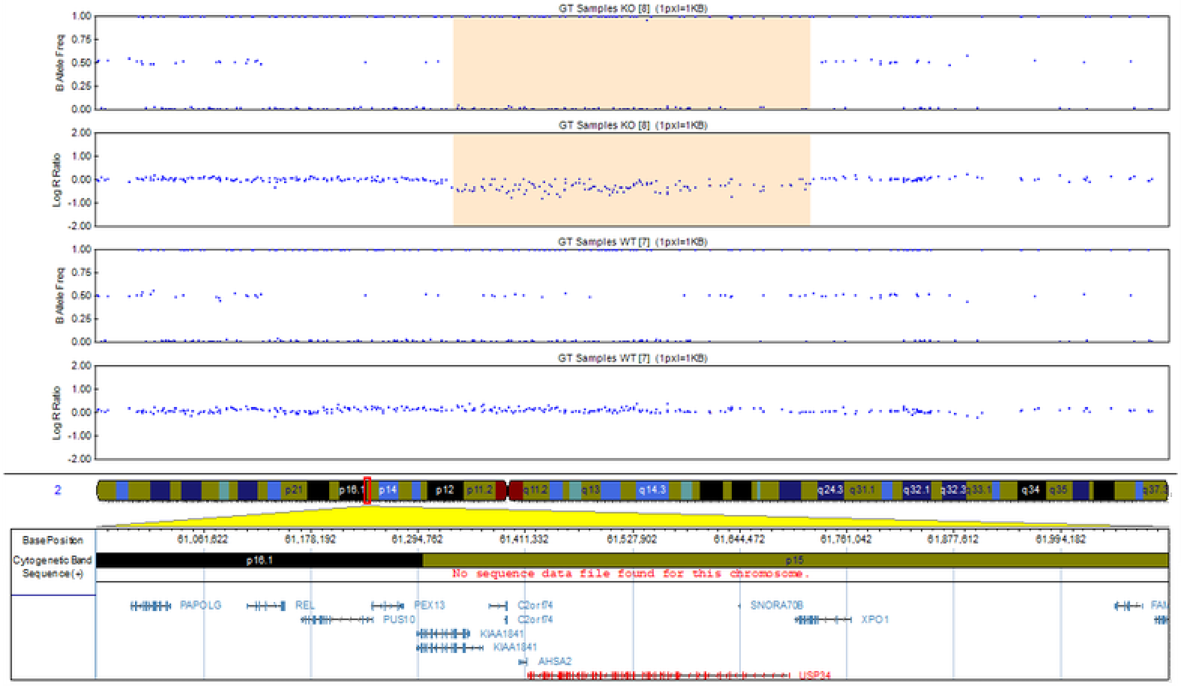

6. In that regard, more data should be provided as to how many passages the hESC line used had undergone, and how different that was in the CRISPR edited line. Most labs would incorporate CRISPR-only (no or irrelevant gRNA) controls with clonal re-expansion to control for the wide difference in passage number between WT and edited lines in this type of experiment. Absent such controls, WGS or an alternative prospective means of establishing post-editing genomic stability (e.g., GuideSeq, etc) should be used.

We have assessed our clones as above and added this as requested. We have added the passage numbers of our cells to the methods section (page 21).

Minor points

1. It's often unclear whether the authors are referring to OPCs, oligodendrocytes, or both. This needs to be clarified throughout the text.

We apologise and have been through the manuscript to ensure this is clear.

2. It's notable that using the Livesey protocol, the cells were caudalized by RA addition. Might this affect their migration biases in vivo in the callosal transplant model? Might OPCs not subjected to high initial RA have a different relative level of Nrp1 expression or different repertoire of Sema3A receptors, and respond differently to Sema3A? I recognize that this is beyond the scope of the authors present work, but it does warrant brief coverage in their Discussion.

We accept this comment and have added to the discussion that we have used one protocol for generation of hES-derived OPCs from one ES cell line and recognise that this is a limitation (page 15).

3. Some verification of the persistence and duration thereof of bioactive Sema3A after tissue injection is needed, especially in the injections into lyso lesions at 48 hrs (as in figure 3). The post-lysolecithin lesion environment is highly proteolytic; how confident are the authors as to the levels and activity of Sema3A that persist?

In our previous publication (Boyd et al., 2011), we addressed this point, which is important. We added the SEMA3A at a timepoint separate from the LPC, partly as we worry that the detergent action of the LPC may interfere with the structure/action of SEMA3A protein, but also as mouse lesions express endogenous SEMA3A for the first 3 days after LPC injection before it is downregulated, which is likely a factor in why mouse demyelinated lesions remyelinated efficiently (Fig. 5, Boyd et al., 2011). We also injected the SEMA3A with laminin, (again as done in Boyd et al., 2011) as SEMA3A is secreted and binds to extracellular matrix and this serves to maintain more persistent expression sufficient to produce poor remyelination (Fig.8, Boyd et al., 2011).

4. In that regard, lysolecithin best models acute demyelination; it is a suboptimal model for the chronic effects of a demyelinating injury, with the astrogliosis, microglial infiltration, and variable axonal loss that attend these lesions. It doesn't necessarily reflect very well the environment into which modified OPCs would be delivered in patients. The authors should discuss these limitations of their model, and also discuss alternative models for focal demyelination (ethidium/RT, short cuprizone, Theiler's, etc.) that might have been used, and their specific reasons for using lysolecithin.

We agree that no model of MS is perfect and have added the use of one chosen model as a limitation of this work in the discussion (page 15). We chose the stereotactic injection of lysolecithin into the corpus callosum as our model as we needed a focal lesion in a predictable location, which allowed for transplantation at a distance and assessment of recruitment. The

LPC model also is very well characterised for the timing of the different steps of remyelination, is safer to the surgeon than ethidium bromide and this is consistent in our hands. We have added this reasoning to the results (page 9).

5**. The authors in several instances state that their work will "reignite" interest and activity in the use of OPC transplantation as a therapeutic strategy. It's not clear to me that such interest was ever extinguished, to be so reignited – this has been, and remains, an active field of investigation across many labs. The authors claim to be reigniting the field seems a bit overstated, and would really best be toned down.**

We have changed this wording (page 15).

We hope that this article will now be deemed suitable for publication in Nature Communications.

Yours sincerely,

Anna Williams and Laura Wagstaff

REVIEWERS' COMMENTS

Reviewer #1 (Remarks to the Author):

In the revised manuscript, the authors added additional scRNAseq characterization of hESC-derived cells, as well as quantification of the identity of these cells following transplantation. Unfortunately, my primary concern with this manuscript, which is that the beneficial effect of NRP1 deletion from OPCs on their capacity to migrate into demyelinated lesions requires the artificial introduction of Sema3A, has not been addressed. As such, I remain unconvinced of the relevance of this approach for improving remyelination in more physiological contexts.

Reviewer #2 (Remarks to the Author):

My concerns have been entirely addressed in this revised version. This strategy of combining gene therapy with OPC transplantation to overcome the cues that prevent remyelination is a wonderful contribution to the field of regenerative medicine in MS. Congratulations!

Reviewer #3 (Remarks to the Author):

The authors have addressed my concerns satisfactorily.
